# Silver Nanoparticle‐Mediated Antiviral Efficacy against Enveloped Viruses: A Comprehensive Review

**DOI:** 10.1002/gch2.202400380

**Published:** 2025-03-28

**Authors:** Ekaterine Mosidze, Gianluigi Franci, Federica Dell'Annunziata, Nicoletta Capuano, Marica Colella, Flora Salzano, Massimiliano Galdiero, Aliosha Bakuridze, Veronica Folliero

**Affiliations:** ^1^ Department of Pharmaceutical Technology 33 Vazha‐Pshavela Ave Tbilisi 0178 Georgia; ^2^ Department of Medicine Surgery and Dentistry “Scuola Medica Salernitana” University of Salerno Via S. Allende 43 Baronissi 84081 Italy; ^3^ Microbiology and Virology Unit, Interdisciplinary Department of Medicine University of Bari “Aldo Moro” Piazza G. Cesare 11 Bari 70124 Italy; ^4^ Department of Theoretical and Applied Sciences (DiSTA) eCampus University Novedrate 22060 Italy; ^5^ Department of Experimental Medicine University of Campania “Luigi Vanvitelli” Naples Italy

**Keywords:** antiviral activity, silver nanoparticles, viral diseases, viral infection, virus

## Abstract

Viral infections continue to pose a significant challenge to global health, with increasing resistance to conventional antiviral therapies highlighting the urgent need for alternative treatment strategies. Silver nanoparticles (AgNPs) have attracted attention as broad‐spectrum antiviral agents due to their unique physicochemical properties and ability to target multiple stages of viral infection. This review provides a comprehensive analysis of the antiviral mechanisms of AgNPs, highlighting their efficacy against clinically relevant enveloped viruses such as influenza, herpes simplex, hepatitis B, and coronaviruses. How key nanoparticle characteristics, including size, shape, surface functionalization, and synthesis methods, influence their antiviral performance is examined. Studies indicate that AgNPs exert their effects through direct interactions with viral particles, inhibition of viral adhesion, and entry into host cells with disruption of viral replication. Furthermore, their potential applications in therapeutic formulations, antiviral coatings, and nanomedicine‐based strategies are explored. Despite their promise, challenges regarding cytotoxicity, stability, and large‐scale production must be addressed to ensure their safe and effective clinical use. This review highlights the transformative potential of AgNPs in antiviral therapy and highlights the need for further investigation to facilitate their clinical translation in the fight against emerging and drug‐resistant viral infections.

## Graphical abstract

Silver nanoparticles (AgNPs) exhibit broad‐spectrum antiviral activity by targeting key viral structures and processes. They disrupt viral envelopes, compromising integrity, and bind to nucleocapsids, impairing viral replication. AgNPs also inhibit glycoprotein interactions, preventing viral attachment and entry into host cells. These multifaceted mechanisms effectively suppress viral infectivity and replication, making AgNPs a promising tool against diverse enveloped and drug‐resistant viruses.

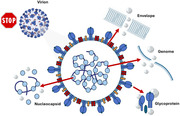

## 1 Introduction

Enveloped viruses are characterized by a lipid‐containing membrane that envelops the virion, in contrast to non‐enveloped viruses, which do not have a membrane encapsulating their protein capsid. These viruses, which are often transmitted via the respiratory tract and body fluids, pose a significant health risk and have been implicated as pathogens in major disease outbreaks and pandemics.^[^ ^1^ ^]^ For example, the 1918 influenza pandemic caused by an influenza virus of the Orthomyxoviridae family^[^ ^2^ ^]^ and Filoviruses such as Ebola and Marburg that led to severe epidemics of hemorrhagic fever. Coronaviruses have been identified as the cause of the SARS outbreak in 2003, the MERS outbreak in 2012, and the current COVID‐19 pandemic.^[^ ^3^ ^]^ The ease of transmission, high replication rates, and mutation potential of enveloped viruses, combined with the lack of broad‐spectrum antiviral therapeutics, indicate that these viruses have significant potential to cause future pandemics and biosecurity threats.^[^ ^4^ ^]^

Various strategies are used to combat enveloped viruses. Prophylactic vaccination is a fundamental tool to prevent viral infections and has led to successful vaccines against many enveloped viruses. However, some viruses evade targeted vaccination, so there are no licensed vaccines for critical human pathogens such as hepatitis C virus (HCV), human immunodeficiency virus (HIV), cytomegalovirus (CMV), and Epstein–Barr virus (EBV).^[^ ^5^ ^]^ Despite an effective vaccine against hepatitis B virus (HBV), ≈240 million people suffer from chronic infection, highlighting the need for curative treatments. This underscores the urgent need for innovative antiviral agents that target enveloped viruses.^[^ ^6^ ^]^

In recent years, efforts in the development of antiviral agents have focused on the use of inhibitory molecules that primarily target viral components to reduce side effects.^[^ ^7^, ^8^ ^]^ However, targeting specific viral elements has drawbacks, including the risk of resistance development and limited application to a single virus. Given the increase in emerging infectious diseases caused by various enveloped pathogenic viruses and increasing antiviral resistance to conventional drugs, pharmaceutical companies and researchers are actively exploring novel antiviral agents.^[^ ^9^ ^]^

Over the last two decades, nanotechnologies have shown great promise in the fight against viruses. Silver nanoparticles (AgNPs) have attracted scientific interest due to their broad spectrum of antimicrobial activity and potential applications in various biomedical fields.^[^ ^10^, ^11^ ^]^ AgNPs are nanoscale particles consisting of silver atoms, typically between 1 and 100 nanometers in size and with a variety of shapes.^[^ ^12^ ^]^ The unique physicochemical properties of AgNPs play a critical role in their biological interactions and applications, with variations in size, shape, and surface properties significantly affecting their biological responses and efficacy.^[^ ^13^ ^]^

The synthesis of AgNPs involves a wide range of techniques, including chemical, physical, and biological methods, each of which imparts specific properties to the nanoparticles. This versatility of synthesis techniques allows researchers to tailor the size, structure, and surface properties of AgNPs, influencing their biological activities and potential applications.^[^ ^14^ ^]^ Extensive evidence demonstrates the potent antibacterial and antifungal properties of AgNPs, particularly their efficacy against common pathogens such as *Staphylococcus aureus*, *Escherichia coli*, and *Candida albicans*.^[^ ^15^ ^]^

Advanced techniques such as high‐resolution microscopy, spectroscopy, and detailed molecular and biochemical analyses have elucidated the mechanisms underlying the antimicrobial effect of AgNPs.^[^ ^16^ ^]^These nanoparticles exert their antimicrobial effect via four different mechanisms: Adhesion to the cell wall and membrane surface, infiltration of cellular structures causing intracellular damage, induction of oxidative stress by ROS and free radicals, and modulation of signal transduction pathways.^[^ ^11^, ^17^ ^]^ The first step involves the binding of AgNPs to the cell wall and membrane of microorganisms. This is facilitated by the positive surface charge of the nanoparticles, which build up an electrostatic attraction to the negatively charged cell wall. This adhesion leads to rupture of the cell wall and damage to the cytoplasm. AgNPs can penetrate microbial cells and disrupt essential cell functions by interacting with cellular structures and biomolecules.^[^ ^18^ ^]^

Within bacterial cells, AgNPs disrupt critical processes such as the respiratory electron transport chain, leading to the formation of ROS and free radicals that damage lipids, proteins, and DNA and ultimately disrupt cell function.^[^ ^19^ ^]^ AgNPs have been shown to affect bacterial cell signaling and inhibit microbial growth by dephosphorylating key bacterial peptide substrates. Recent studies have highlighted the antiviral potential of AgNPs against a range of virus families, exhibiting direct virucidal activity or altering virus‐host cell binding.^[^ ^20^ ^]^ In 2005, Elechiguerra et al. first described the anti‐HIV effect of AgNPs, paving the way for further investigations into the broad spectrum of antiviral properties of these nanoparticles^.[^ ^21^ ^]^ Despite the well‐established antimicrobial properties, the specific antiviral activities of AgNPs are still relatively unexplored. This review aims to comprehensively explore the antiviral activities of silver nanoparticles and improve our understanding of their mechanisms against various coated viral species with important clinical implications.

## 2 AgNPs and Antiviral Activity

AgNPs have come into focus for their remarkable antimicrobial potential. Despite this well‐documented antibacterial efficacy, the interaction between AgNPs and viruses was neglected until recent scientific efforts brought to light their promising antiviral activity.^[^ ^22^ ^]^ These burgeoning studies have revealed the potential of AgNPs as effective antiviral agents, especially against enveloped viruses.^[^ ^23^ ^]^ The need to explore the interactions of AgNPs with viruses stems from the ease with which enveloped viruses are transmitted, their high rates of replication and mutation, and the current lack of comprehensive broad‐spectrum antiviral therapies.^[^ ^22^ ^]^ The importance of this study is further emphasized by the emerging threat of enveloped viruses contributing to impending pandemics and biosafety challenges. The potential role of AgNPs as a versatile and comprehensive therapeutic option against enveloped viruses takes center stage and offers a promising avenue to fill critical gaps in the currently available antiviral arsenal.^[^ ^23^, ^24^ ^]^

### 2.1 Influenza Viruses

**Figure 1:**
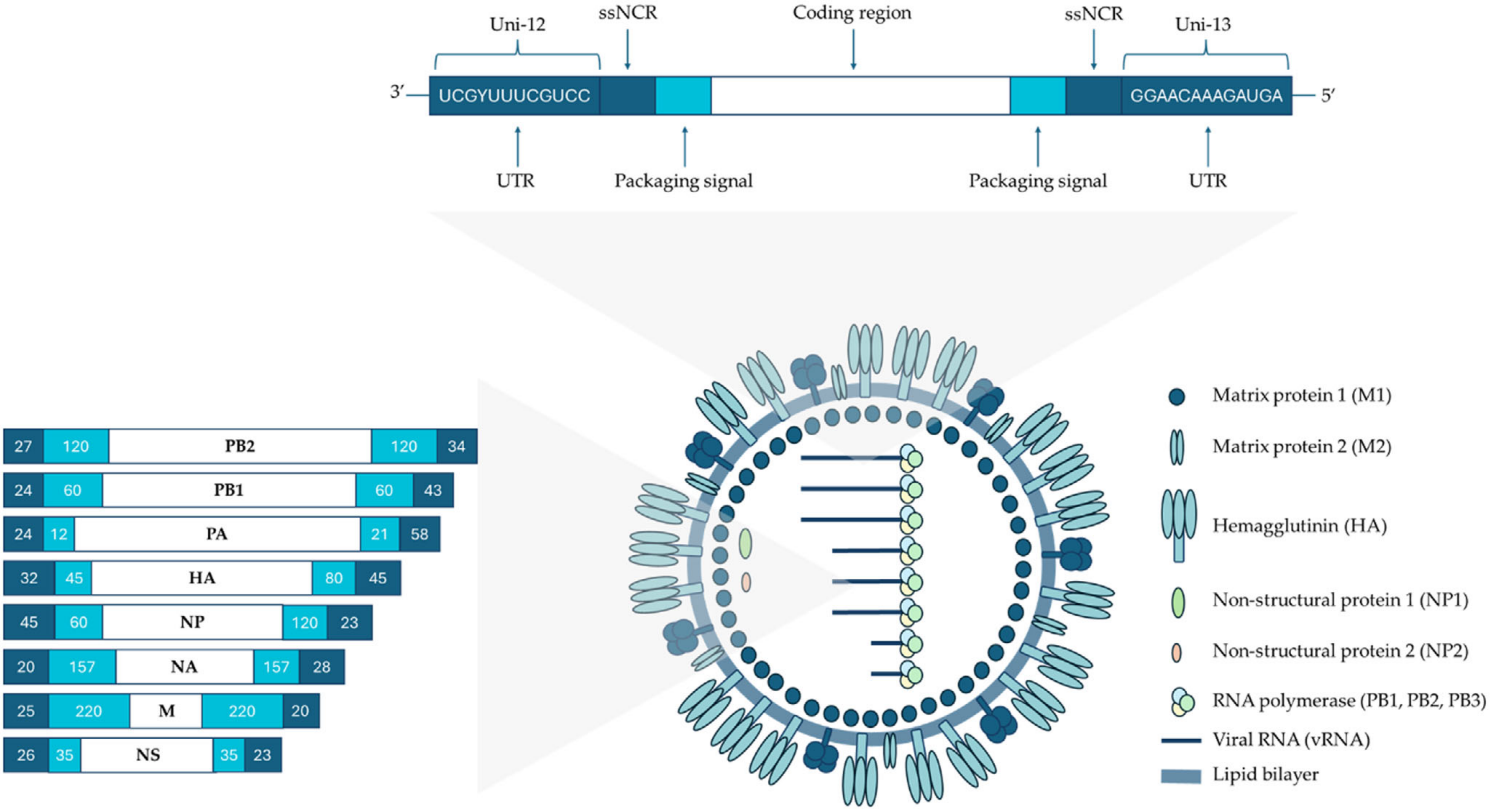
Influenza A virion structure and genome organization.

The influenza viruses belong to the family Orthomyxoviridae, which includes the genera influenza virus type A, influenza virus type B, influenza virus type C, Thogotovirus, Quaranjavirus, and Isavirus.^[^ ^25^ ^]^ These enveloped viruses have a helical capsid and a segmented genome consisting of negative‐sense RNA, encoding proteins vital for viral replication and assembly.^[^ ^25^, ^26^ ^]^ The heterotrimeric polymerase complex (PB1, PB2, PA), nucleoprotein (NP), matrix protein 1 (M1), non‐structural protein (NS1), and nuclear export protein (NEP) are localized within the lipid envelope, while matrix protein 2 (M2), hemagglutinin (HA), and neuraminidase (NA) are integrated into the envelope architecture.^[^ ^27^ ^]^ Hemagglutinin (HA) is a homotrimeric protein that binds to sialic acid on host cells to initiate viral entry via endocytosis or micropinocytosis.^[^ ^28^, ^29^ ^]^ Neuraminidase (NA) is a tetrameric enzyme that helps viruses spread by hydrolyzing sialic acid residues on infected cells and new virions, promoting viral migration and release from host cells.^[^ ^30^ ^]^ Influenza virus M2 is a transmembrane protein that forms a proton channel, regulating pH during viral entry and maturation. The matrix protein M1 under the envelope is essential for virion assembly, linking the capsid and envelope proteins.^[^ ^31^ ^]^ In influenza virions, each segment of viral RNA (vRNA) associates with NEP and the viral polymerase subunits (PB1, PB2, PA) to form ribonucleoprotein particles (RNPs) crucial for replication and transcription. NEP protects single‐stranded RNA and aids in RNP assembly, while NS1 regulates viral protein expression by binding to host and viral RNA. The vRNA is transported to the nucleus for replication and transcription, with viral mRNAs returning to the cytoplasm for translation. Mature genomic segments are packaged into new virions, and neuraminidase (NA) releases them by breaking sialic acid bonds on host cells.^[^ ^32^, ^33^, ^34^, ^35^ ^]^ Among the orthomyxoviruses, influenza A plays a fundamental role, characterized by its epidemiological and clinical importance (**Figure** **1**). In its most severe form, the infection leads to clinically significant manifestations that can be fatal. This underlines the serious impact of influenza A on public health.^[^ ^36^ ^]^ A particular feature of influenza A is its remarkable ability to drift and shift antigens.^[^ ^37^ ^]^ This genetic adaptability enables the virus to make subtle or dramatic changes to its surface proteins. This dynamic ability contributes significantly to the emergence and development of pandemics, which further emphasizes the need for new therapeutic strategies with a broad spectrum of activity. Numerous studies have documented the antiviral activity of AgNPs against various strains of the influenza A virus.^[^ ^38^ ^]^ The earliest available study reporting the antiviral potential of AgNPs dates back to 2009. Mehrbod et al. obtained liquid Nanosilver with 2000 ppm (particle mL^−1^) to test its antiviral activity against Influenza A/New Caledonia/20/99 (H1N1) standard virus.^[^ ^39^ ^]^ At concentrations below 1 µg mL^−1^, the nanoparticles demonstrated nontoxicity to MDCK (Madin‐Darbey Canine Kidney) cells. Moreover, their interference in the adsorption/penetration phase effectively reduced viral infectivity at these nontoxic concentrations. To investigate the target molecules of AgNPs, an evaluation of the ability of antibodies to recognize viral glycoproteins (especially HA and NA) after treatment with the nanoparticles was performed. The results showed that AgNPs hindered the interaction between glycoproteins and antibodies, as evidenced by a decrease in fluorescence after treatment with AgNPs.^[^ ^39^ ^]^ Dong‐xi et al. synthesized AgNPs with an average diameter of 10 nm to perform hemagglutination inhibition assays, an embryo inoculation assay, and a 3‐(4,5‐dimethylthiazol‐2‐yl)‐2,5‐diphenyltetrazolium bromide (MTT) assay.^[^ ^40^ ^]^ These tests were used to evaluate the inhibitory effect of AgNPs against the influenza A H1N1 virus. The results provide convincing evidence for the rapid inhibitory effect of AgNPs on chicken red blood cell hemagglutination in HAI assays and in an embryo inoculation assay against influenza A H1N1 virus.^[^ ^40^ ^]^ In addition, these nanoparticles showed minimal cytotoxicity on MDCK cells at concentrations below 25 µg mL^−1^ after exposure. When evaluating the efficacy of AgNPs against influenza A H1N1 virus, MDCK cells were treated with a combined mixture of influenza A H1N1 virus and silver nanoparticles at non‐toxic concentrations. The survival percentages of MDCK cells at a concentration of 12.5 µg mL^−1^ were 47.97% (72 h) and 49.87% (96 h), while at 25 µg mL^−1^ of AgNPs the percentages were 82.4% (72 h) and 86.5% (96 h). The same research group investigated the potential of AgNPs to inhibit influenza virus‐induced apoptosis in MDCK cells.^[^ ^40^ ^]^ The morphological changes and ultrastructural characteristics of the cells were examined by transmission electron microscope and flow cytometry analysis. The apoptosis rate in normal cells was 5.58 ± 2.20%, while virus‐infected cells controlled a rate of 23.37% ± 2.50%. When cells were treated with the combined mixture of viruses and AgNPs they showed a reduced apoptosis rate of 9.77% ± 1.59%. In contrast, cells treated first with the virus and then with AgNPs had an apoptosis rate of 14.66% ± 1.80%, while those treated first with silver nanoparticles and then with the virus showed an apoptosis rate of 18.48% ± 0.98%.^[^ ^40^ ^]^ In this study AgNPs demonstrated a rapid and effective inhibition of influenza A H1N1 virus, highlighting promising antiviral properties and significant cellular tolerability.^[^ ^40^ ^]^ In a study conducted by Mori et al., composite materials comprising silver nanoparticles (AgNPs) and chitosan (Ch) were synthesized to modulate the infectivity of human influenza A virus (A/PR/8/34 H1N1).^[^ ^41^ ^]^ AgNPs suspensions were produced through the reduction of silver‐containing glass powder (BSP21, silver content 1% by weight, average grain size 10 µm) in an aqueous glucose solution, generating AgNPs suspensions with distinct diameters such as 3,5, 6.5, and 12.9 nm. Subsequently, a chitosan solution (100 µL, 10 mg mL^−1^) was combined with solutions containing AgNPs of various diameters, obtaining nanocomposites characterized by different quantities and diameters of AgNPs.^[^ ^41^ ^]^ The accurate synthesis of the components was evaluated through the analysis of UV‐visible spectra, where the absence of a peak near 400 nm indicated their complete binding to chitosan. Transmission electron microscope analyses confirmed the absence of aggregation of the suspensions.^[^ ^41^ ^]^ After the characterization of the different suspensions, the studies focused on their antiviral activity, comparing the TCID_50_ ratios of the viral suspensions treated and untreated with the nanomaterials. The results demonstrated an increase in the antiviral activity of the AgNPs/Ch composites with increasing nanoparticle content. Chitosan alone did not show antiviral activity, underlining that the observed effect was attributable to the bound AgNPs.^[^ ^41^ ^]^ Furthermore, the impact of the size of the Ag NPs in the composites was evident, with generally stronger antiviral activity observed in composites containing smaller AgNPs at similar concentrations.^[^ ^41^ ^]^ Nanoparticles can enhance drug efficacy due to their microscopic size and unique properties. A key reason for associating them with drugs is their ability to facilitate targeted delivery to diseased cells, improving treatment precision and minimizing side effects on healthy tissues. Additionally, nanoparticles can improve drug solubility and stability, increase the drug's residence time in the body, and enable controlled and sustained release of the active ingredient.^[^ ^42^ ^]^ In this context, Li et al. cleverly used AgNP‐conjugated amantadine to combat the emergence of drug‐resistant influenza viruses. Amantadine is an FDA‐approved antiviral drug, its irregular use has led to the selection of resistant viral strains with mutations in the M2 protein.^[^ ^43^ ^]^ To address this problem, Li and the research team synthesized amantadine‐linked AgNPs (Ag@AM) through a precise procedure. They incrementally introduced 0.1 mL of vitamin C stock solution into 4 mL of AgNO_3_ stock solution, followed by the addition of 0.8 mL of amantadine (1 µm). Overnight dialysis removed excess components, and the resulting Ag@AM nanoparticles were characterized using various techniques.^[^ ^43^ ^]^ The synthesized Ag@AM showed a uniform and monodisperse spherical shape as evidenced by transmission electron microscope images, Zeta potential, and size distribution using Zetasizer Nano ZS. Energy‐dispersive X‐ray spectroscopy (EDX) analysis showed the presence of signals coming from C atoms (22%) which demonstrated the conjugation of AgNPs with amantadine. In interaction studies with H1N1, Ag@AM demonstrated a disruptive effect on viral particles. Transmission electron microscopy revealed the destruction of H1N1 viral morphology after a 30‐min interaction.^[^ ^43^ ^]^ In support, hemagglutination inhibition assays further indicated the ability of Ag@AM to prevent influenza virus‐induced aggregation of chicken erythrocytes, highlighting its potential to inhibit interactions with MDCK cells. Pre‐treated viral particles were used to infect MDCK monolayers. The MTT assay demonstrated the protective effects of Ag@AM on cells against the H1N1 influenza virus. The virus led to a significant decrease in cell viability (39%), both amantadine and AgNPs showed an improvement (56% and 65%, respectively). Surprisingly, Ag@AM increased cell viability by up to 90%, demonstrating its superior protective capabilities. Further investigation using JC‐1 dye revealed that Ag@AM mitigated H1N1 virus‐induced mitochondrial dysfunction, in contrast to the effects of amantadine and AgNPs.^[^ ^43^ ^]^ Flow cytometric and TUNEL‐DAPI analyses demonstrated a notable reduction in the apoptotic cell population (13.5%) with post‐viral Ag@AM infection. In the context of influenza virus infections, the emergence of excess reactive oxygen species (ROS) often coincides with the onset of cellular apoptosis. To discern whether the combined nanoparticles possess the ability to prevent ROS‐mediated apoptosis induced by the H1N1 virus, intracellular ROS levels were monitored through fluorescence intensity examination, specifically focusing on dichlorofluorescein (DCF). Data indicated a decrease in intracellular ROS generation, further supporting the antiviral potential of Ag@AM. Taken together, these data offer promising results in the inhibition of the H1N1 influenza virus.^[^ ^43^ ^]^ The same group of researchers exploited the same strategy to combat the emergence of oseltamivir‐resistant influenza viruses. They conjugated AgNP with oseltamivir (Ag@OTV), according to the same procedure indicated previously, and evaluated the antiviral mechanism. The synthesized Ag@OTV exhibited a spherical shape as observed through transmission electron microscope images. Analysis of Zeta potential and size distribution using Zetasizer Nano ZS further supported the monodisperse nature of the particles. EDX analysis revealed signals from C atoms (18%), indicating the successful conjugation of AgNPs with oseltamivir.^[^ ^44^ ^]^ In interaction studies with H1N1, transmission electron microscopy revealed an alteration of H1N1 viral morphology, preventing aggregation of chicken erythrocytes and consequently influencing interactions with MDCK cells. Particles pretreated with Ag@OTV increased cell viability by up to 90% in response to viral infection.^[^ ^44^ ^]^ On the other hand, the virus caused a significant decrease in cell viability (39%), while oseltamivir and AgNP showed improvements (59% and 65%, respectively), which were lower than the combined counterparts. Further investigation using JC‐1 dye demonstrated that Ag@OTV mitigated H1N1 virus‐induced mitochondrial dysfunction. Flow cytometric and TUNEL‐DAPI analyses demonstrated a notable reduction (14.6%) in the apoptotic cell population in response to Ag@OTV treatment. This effect is a result of the nanostructure's ability to reduce ROS production and inhibit caspase 3. Furthermore, Li et al. proposed a strategic approach to overcome drug resistance in influenza A H1N1 infections.^[^ ^43^ ^]^ Zanamivir represents one of the most effective drugs against influenza virus infection. Unfortunately, its use is limited due to the emergence of resistant influenza viruses. Also, in this case, AgNPs were exploited to improve the antiviral potential. In the study conducted by Lin et al., AgNPs were conjugated with Zanamivir, resulting in the formation of Ag@ZNV.^[^ ^45^ ^]^ The resulting Ag@OTV particles exhibited a spherical morphology, uniform, monodisperse, and non‐aggregated distribution, as indicated by transmission electron microscope and Zetasizer Nano ZS investigations. EDX analysis revealed signals from Ag (59.5%), C (23%), N (8.6%), and O (8.9%) atoms, indicative of successful drug conjugation reaction. AgNP, ZNV and Ag@ZNV showed a dose‐dependent decrease in cell viability, with concentrations of 2.5 µg mL^−1^, 2.5 µg mL^−1^, and 0.8 nm, respectively. The viability of MDCK cells during H1N1 virus infection was 36.32%. Zanamivir and AgNP treatments increased the viability to 63.23% and 61.87%, while Ag@ZNV significantly reached 82.26%. The increased cell viability in response to infection was due to the interference of NA activity^[^ ^46^ ^]^ (**Table** **1**).

**Table 1:** Characteristics and Activity of AgNPs Against Influenza Viruses.

Type of nanoparticle	Synthesis method	Viruses	Active concentrations	Refs.
AgNPs and Chitosan	A mixture of 0.50 g silver‐containing glass powder and 1.00 wt% glucose solution was heated in an autoclave at 121 °C and 200 kPa for 20 minutes. After cooling, it was centrifuged, and the supernatant was collected and stored in the dark at room temperature.	H1N1	≤ 200 µg AgNPs/g Chitosan	[41]
AgNPs (Ag@AM) Amantadine linked to AgNPs	Chemical methods	H1N1	2.5 µg mL^−1^, AgNPs, 0.1 µm AM	[43]
AgNPs conjugated with Oseltamivir (Ag@OTV)	Biological method	H1N1	2.5 µg mL^−1^ AgNPs, 1 nm OTV	[44]
AgNPs conjugated with Zanamivir (Ag@ZNV)	Biological method	H1N1	2.5 µg mL^−1^, 25 µg mL^−1^ and 0.8 nm	[45]
AgNPs and FluPep ligand	AgNPs of approximately 10 nm in diameter were acquired from nanoComposix Inc. (CA, USA)	H1N1	From 0.03% to 5% mol mL^−1^ of conjugated ligand	[47]
AgNPs	Cinnamon bark was finely ground and 25 g of the powder was combined with 100 mL of deionized water. After filtering the extract, 10 mL was mixed with 90 mL of a 1 mm silver nitrate solution. The mixture was left at room temperature for 5 h, during which the formation of silver nanoparticles (AgNPs) was indicated by a color change.	H7N3	125 µg mL^−1^	[48]
AgNPs with tannic acid	Tannic acid, AgNO3 and NaOH solutions were prepared, mixed and stirred to synthesize AgNps. The mixture was adjusted to pH 9, stirred for 30 min and centrifuged to isolate the product, which was washed with water. AgNPs were applied to the cotton fabric using a drop‐coating method. Then the fabric was dried at 60 °C with varying concentrations of AgNps	H3N2	–	[49]
AgNPs	Biological method	H5N1	60 µm of EGCG concentration	[50]
EGCG‐AgNPs together with zinc sulfate	Chemical method	H9N2	50 µm EGCG resulted in the reduction in logEID50 mL^−1^ of AI H9N2	[50]
AgNPs with amino acid conjugate	Silver nitrate was combined with an amino acid conjugated to Tris(hydroxymethyl)phosphine, and the mixture was placed in a water bath sonicator at 80 °C for 24 h.	H1N1	17 µg mL^−1^	[51]
Ag NPs immobilized on cotton fabrics	AgNps were produced through a radiochemical synthesis process.	H1N1	–	[52]
Immobilization of AgNPs on resin substrates	AgNps were produced through a radiochemical synthesis process.	H3N2	–	[53]
AgNPs	Purchased from Noble Elements LLC, USA	H1N1	From 4 to 2000 µg mL^−1^	[54]
AgNPs	AgNPs were created by combining silver nitrate, sodium carbonate, and tannic acid in distilled water, with the pH adjusted to 7.4. The mixture was refluxed and stirred for 2 h until the pale yellow color remained constant, indicating successful AgNP formation.	H3N2	From 50 to 12.5 µg mL^−1^	[55]
AgNPs	AgNPs were produced by reducing silver nitrate with hydrazine hydrate, using potassium oleate as a stabilizing agent.	H1N1	0.2 mg of atomic silver per 1 kg body mass	[46]

### 2.2 Severe Acute Respiratory Syndrome Coronavirus 2 (SARS‑CoV‑2)

**Figure 2:**
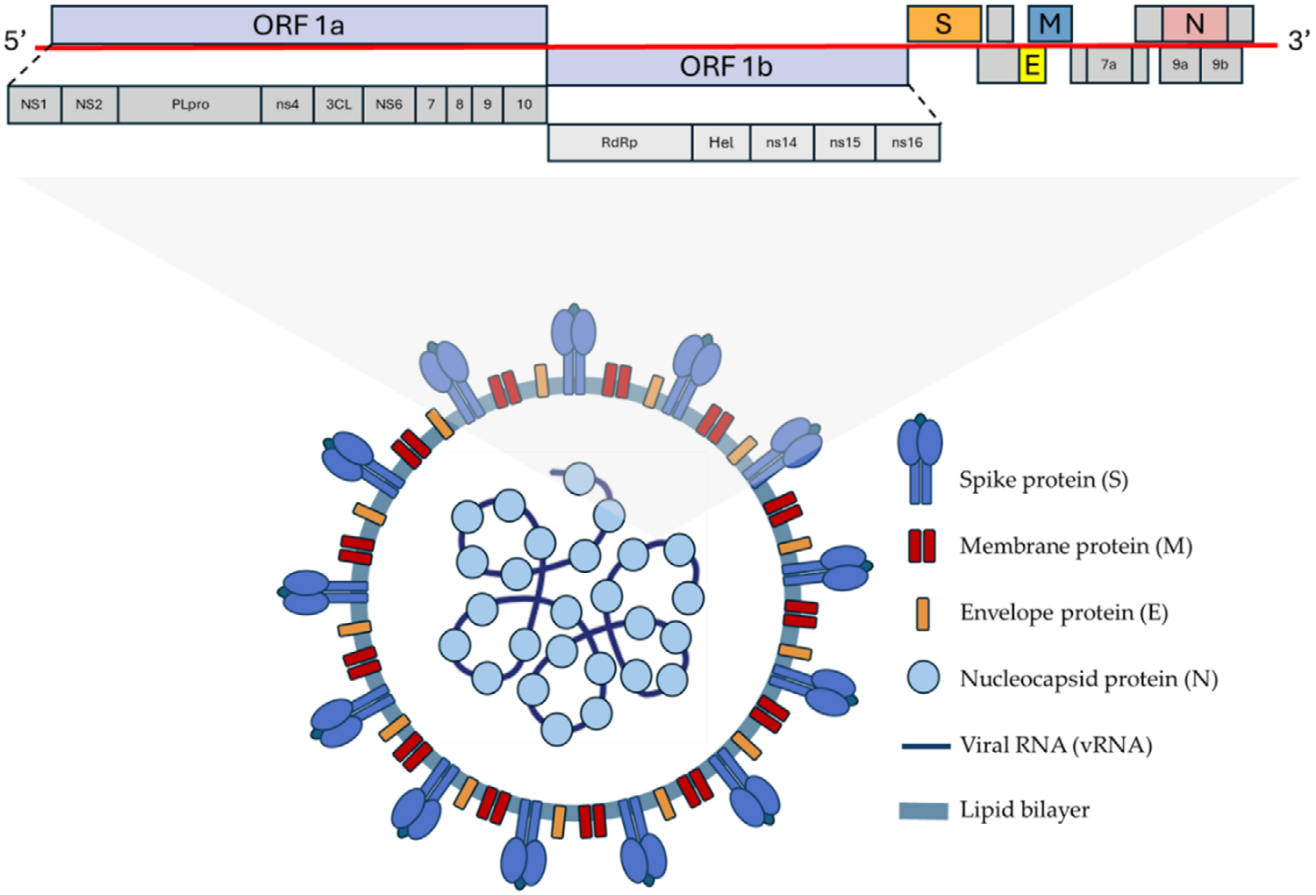
SARS‐CoV‐2 virion structure and genome organization.

The spread of infection caused by SARS‐CoV‐2 was declared a pandemic by WHO on March 11, 2020. SARS‐CoV‐2 infected millions of people due to its extremely contagious nature and capacity to remain active on surfaces for a prolonged period. SARS‐CoV‐2 has a diameter of roughly 100 nm and has a spherical shape. It is a member of the beta‐coronavirus family and is a single‐stranded positive‐strand RNA virus with an envelope, coated with a lipid wrap produced from host cell membranes. SARS‐CoV‐2 employs its highly glycosylated homotrimeric spike (S) protein, a class I viral fusion protein, for cellular entry. This spike protein comprises S1 and S2 subunits. The S1 subunit contains the receptor binding domain (RBD), which binds to the host angiotensin‐converting enzyme 2 (ACE2).^[^ ^56^ ^]^ Upon binding to ACE2, an additional site within the S2 subunit, termed S2′, becomes exposed. Cleavage of S2′ by host transmembrane serine protease 2 (TMPRSS2) at the plasma membrane or by cathepsin L in the endosomal compartment following ACE2‐mediated endocytosis initiates a series of folding events in S2,^[^ ^56^ ^]^ leading to the release of hydrophobic fusion peptide (FP) and facilitating membrane fusion. The ingress of the coronavirus genome into the cytoplasm of the host cell initiates a sophisticated cascade of events governing viral gene expression.^[^ ^57^ ^]^ Upon cellular entry, the viral RNA genome undergoes prompt translation, yielding pivotal effector molecules that orchestrate the viral replication cycle. Structural proteins, including M and E, undergo intricate trafficking processes to attain maturity, ultimately localizing to the plasma membrane or integrating into virions. Within the cytoplasm, nonstructural proteins (NSPs) such as NSP7, NSP8, NSP12, and NSP13 assemble into the RNA‐dependent RNA polymerase (RdRp) complex, essential for synthesizing the viral genome. Concurrently, viral proteases, exemplified by NSP3 and NSP5 (also termed papain‐like protease, PLpro, and major protease, Mpro, respectively), not only facilitate the maturation of viral components from polyproteins but also cleave host factors, thus subverting innate immune responses within target cells. Furthermore, certain effectors target cellular membrane organelles, perturbing normal homeostatic processes to foster viral replication. For instance, Orf9b selectively interacts with the mitochondrial membrane protein TOM70, suppressing the activity of the mitochondrial antiviral signaling protein (MAVS) (**Figure** **2**).^[^ ^58^ ^]^

One strategy to mitigate the transmission of the virus involves the inactivation of viral particles during their transit on surfaces. This approach diminishes the potential for infection spread and can be accomplished by implementing antiviral coatings on possible fomites.^[^ ^59^ ^]^ These coatings, often formulated with various metals, exhibit virucidal properties, thereby reducing the viability of the virus upon contact. Notably, such coatings can be applied to protective gear such as face masks, enhancing their efficacy as a barrier against viral transmission. Silver nanoparticles (AgNPs) have attracted considerable attention for their potential usefulness in combating viral transmission and multiple research projects have been conducted in functionalized fabrics or surfaces.^[^ ^60^ ^]^

Kumar et al. conducted a study to assess the antiviral efficacy of fabrics treated with silver nanoparticles (AgNPs) against SARS‐CoV‐2.^[^ ^61^ ^]^ Initially, cotton fabric underwent impregnation with a solution comprising acetone and distilled water, followed by drying. Subsequently, the fabric was immersed in a 1 mm AgNO_3_ solution and exposed to UV light (254 nm) for 30 min. After drying at 50 °C, the fabric's color changed from white to dark brown, indicating successful AgNP integration. To evaluate the virucidal activity, the treated fabrics were co‐incubated with viral inoculum (0.1 MOI) for 30 and 60 min, with non‐functionalized fabrics serving as controls. Following incubation, the viral inoculum was introduced to Vero cell monolayers and allowed to incubate for 3 h. Subsequently, the fresh medium replaced the viral inoculum, maintaining the cells for up to 72 h. Post‐infection, plaque assays, and RT‐PCR were employed to assess the presence of virions and viral genes in the cells. The findings demonstrated significant virucidal activity of the modified fabric against SARS‐CoV‐2, exhibiting a remarkable 97% inhibition of the viral load employed in the assays.^[^ ^61^ ^]^ Balagna et al. investigated the efficacy of face masks coated with silver nanoclusters against SARS‐CoV‐2.^[^ ^62^ ^]^ The coating process utilized radio frequency co‐sputtering in a pure argon atmosphere to deposit a silver nanocluster/silica‐based coating onto two disposable FFP3 face masks (3M). This procedure involved applying 3 W or 5 W of DC power to the silver target, resulting in coatings with varying concentrations of silver nanoclusters, designated as Ag3 W and Ag5W. Two experiments were conducted to assess the antiviral activity of the coated face masks against SARS‐CoV‐2. In the initial experiment, viral inoculum was introduced to both coated and uncoated mask samples, followed by incubation at room temperature. Both sets of specimens underwent sterilization to prevent contamination. The second experiment was similar but omitted sterilization to mimic real‐world conditions. Following incubation, virus infectivity was evaluated by adding it to Vero cells in a microtiter plate and observing cytopathic effects and viable cell staining after 72 h of incubation. Results revealed that uncoated face masks and control samples exhibited the highest infectivity. Conversely, the coated masks demonstrated a significant reduction in infectivity, with the Ag3 W coating showing a substantial decrease and the Ag5 W coating eliminating cytopathic effects.^[^ ^62^ ^]^ Estevan et al. assessed safety considerations associated with the utilization of masks incorporating nanoparticle functionality. The investigation focused on internal exposure levels stemming from the utilization of face masks coated with silver nanoparticles (AgNPs). Results indicated that internal exposure levels ranged from 7 × 10‐5 to 2.8 × 10‐4 mg kg^−1^ bw^−1^ d^−1^. These findings suggest that prolonged exposure to AgNPs arising from the utilization of face masks is deemed safe.^[^ ^63^ ^]^ Baselga et al. have developed a new antiviral coating designed to improve the effectiveness of surgical masks and materials used in air conditioning filters.^[^ ^64^ ^]^ This virucidal coating incorporates AgNPs (6.2 nm diameter) at a concentration of 5 mg mL^−1^, along with polyethyleneimine (PEI) at 10 mg mL^−1^. This combination determines the formation of a uniform and homogeneous coating, which extends across the entire surface and depth of the material. To evaluate the efficacy of the functionalized tissues, they were exposed to a viral inoculum with a multiplicity of infection (MOI) of 0.01. Subsequent evaluation of cell monolayers revealed a viral load reduction of more than 99.9% after incubation. Furthermore, the cytotoxicity of the coatings was evaluated using Vero E6 cells, indicating that no cytotoxic effects were observed. In particular, the inclusion of PEI was found to mitigate the toxicity associated with nanoparticles, thus improving the safety profile of the coating.^[^ ^64^ ^]^ The factors influencing the effects demonstrated by silver nanoparticles are of considerable scientific interest. These factors encompass a wide array of parameters, including the size, shape, surface properties, concentration, and surrounding environment of the nanoparticles. Jeremiah et al. have conducted research elucidating the relationship between the virucidal efficacy of silver nanoparticles (AgNPs) and their size. Specifically, AgNPs with diameters of 2, 15, 50, 80, and 100 nm underwent antiviral assessments using plaque assays for SARS‐CoV‐2 on VeroE6/TMPRSS2 and Calu‐3 cell lines. Additionally, cytotoxicity was evaluated utilizing the CellTiter‐Glo test across concentrations ranging from 1 to 100 ppm on the same cell lines. Results indicated that nanoparticles exhibited cytotoxic effects at concentrations exceeding 20 ppm. Notably, antiviral assays revealed that nanoparticles ≤10 nm in size demonstrated maximal antiviral efficacy within concentrations ranging from 1 to 10 ppm. This research underscores the size‐dependent nature of AgNP virucidal efficacy and provides valuable insights for optimizing their antiviral properties while mitigating cytotoxicity concerns.^[^ ^65^ ^]^ In a study conducted by He et al., three distinct surface coatings—citrate, polyvinylpyrrolidone (PVP), and branched polyethylenemine (BPEI)—were selected as modification materials for silver nanoparticles (AgNPs) to assess their virucidal effects against SARS‐CoV‐2 in vitro.^[^ ^66^ ^]^ Ten different types of AgNPs were examined, with sizes ranging from 5 to 100 nm for citrate and PVP, and 50 to 100 nm for BPEI. Flow cytometry and the CCK‐8 method were employed to evaluate apoptosis and cell viability, respectively. To assess the safety and anti‐SARS‐CoV‐2 impact of each AgNP variant in vitro, quantitative reverse transcription‐polymerase chain reaction, Hoechst staining, crystal violet staining, and virus titration were conducted. Results indicated that AgNP treatment significantly enhanced viability by up to 20.70%, whereas SARS‐CoV‐2 infection led to a significant decrease in viability. The efficacy of AgNPs varied based on nanoparticle size and surface modification. Specifically, smaller AgNPs (5, 20, 50, and 100 nm) exhibited more pronounced effects in enhancing viability. A similar trend was observed regarding the reduction of viral replication by AgNP treatment, wherein smaller nanoparticles demonstrated a greater reduction in viral titer, underscoring a size‐dependent inhibitory effect.^[^ ^66^ ^]^ Merkl et al. investigated a surface modification strategy targeting enveloped SARS‐CoV‐2 through aerosol nanoparticle self‐assembly during the flame synthesis of three antimicrobial materials: silver (Ag), copper oxide (CuO), and zinc oxide (ZnO).^[^ ^67^ ^]^ Their antiviral efficacy against SARS‐CoV‐2 viability was assessed via a viral plaque assay utilizing Vero‐E6 cells. Among the three studied nanomaterials, nanosilver particles exhibited the strongest antiviral activity. Following 5 min of exposure, there was a 75% reduction in viral load, which further increased to a 98% reduction after 120 min of exposure. Transmission electron microscopy (TEM) analysis revealed that the average primary nanosilver particle diameter was ≈5 nm.^[^ ^67^ ^]^ Similar to previous work, Asmat et al. conducted research aimed at developing antiviral fabrics impregnated with silver nanoparticles (AgNPs), zinc oxide, and cuprous oxide to combat SARS‐CoV‐2.^[^ ^68^ ^]^ The team employed a biogenic synthesis approach, recognized for its ability to generate nanostructures with specific morphology, high resistance to degradation, and monodispersity, offering a promising alternative in nanostructure fabrication. Fabrics were impregnated using both “in situ” and “post‐synthesis” methods. For the biogenic production of silver nanoparticles, a 96% alcoholic extract of *Eucalyptus globulus* was utilized, resulting in AgNPs with a diameter of 6 nm. Notably, the “in situ” treatment demonstrated superior nanoparticle impregnation, with nanostructures observed in both the core and surface of the textile fiber, whereas the “post‐synthesis” method led to agglomerates primarily on the surface. To assess antiviral activity, SARS‐CoV‐2 E gene RNA was extracted, quantified, and subjected to PCR reaction. Results indicated that AgNPs, while not the most effective among the tested materials, demonstrated a significant reduction in viral load. Specifically, the “in situ” treatment achieved a 95.56% reduction, while the “post‐synthesis” method exhibited an 88% reduction with a concentration of 2.41 ppm.^[^ ^68^ ^]^ Lam et al. introduced the first polyurethane (PU)/silver nanoparticle (AgNP) composites, demonstrating promising coating properties.^[^ ^69^ ^]^ The researchers designed and synthesized the PU‐based composite using a multigram‐scale one‐pot synthesis procedure from commercially available starting materials. When loaded with 10 wt% silver nanoparticles, PU formed a durable composite thin film. The incorporation of AgNPs influenced the mechanical properties of the composite, resulting in increased tensile strength, toughness, and elasticity modules, albeit with a reduction in elongation at break compared to pristine PU. Comparative bioassays were conducted by applying a pseudotyped virus (PV) bearing the SARS‐CoV‐2 beta (B.1.351) spike protein onto the film surfaces of pristine PU or PU nanocomposite. Within 24 h, the nano complexes exhibited a significant reduction in viable virus compared to pristine PU under the same conditions, with a 67% decrease (*p* = 0.0012). This highlights the potential antiviral efficacy of the PU/AgNP composite as a coating material against SARS‐CoV‐2.^[^ ^69^ ^]^ Diaz‐Puertas et al. incorporated commercially available ceramic‐coated silver nanoparticles (AgNPs) into thermoplastic polyurethane (TPU) plates using an industrial‐scale protocol.^[^ ^70^ ^]^ The embedded nanoparticles were characterized to have a size of 54.4 nm. Through ICPMS analysis, the release rate of silver from AgNP‐TPU in aqueous solvents was determined to be 4 ppm h^−1^. Biological tests conducted on Vero E6 and EPC cell lines revealed that the AgNP‐TPU material did not induce significant cytotoxicity. Additionally, AgNP thermoplastic polyurethane (TPU) demonstrated 75% activity against SARS‐CoV‐2 in a time‐ and temperature‐dependent manner. These findings suggest that AgNP‐TPU‐based materials hold promise as an effective strategy to inhibit viral propagation.^[^ ^70^ ^]^ Da Silva et al., successfully prepared PVC/AgNP nanocomposites through melt mixing, a suitable method for large‐scale production of polymeric products.^[^ ^71^ ^]^ Characterization of the obtained composite revealed that the addition of AgNPs improved the material's thermal and mechanical characteristics and more importantly, PVC/AgNP nanocomposites, well‐established antimicrobial compounds, have virucidal action against SARS‐CoV‐2, The antiviral assay tested various contact times (30, 60, and 120 min), revealing that longer durations result in higher viral inactivation rates. PVC/0.3AgNP and PVC/0.5AgNP samples exhibited significant virucidal activity compared to cell controls and SARS‐CoV‐2 positive controls according to Dunn's tests. It requires at least 0.3 wt% of AgNPs to achieve a 99.99% inactivation rate within 48 h.^[^ ^71^ ^]^ Data on the antiviral efficacy of AgNPs against SARS‐CoV‐2 are not limited to in vitro experiments but extend to in vivo studies as well. Almanza‐Reyes et al. conducted a study at the General Hospital of Tijuana, Mexico, a facility dedicated exclusively to COVID‐19 patients, to investigate the effectiveness of mouthwash and nasal rinse containing silver nanoparticles (ARGOVIT AgNP) in preventing SARS‐CoV‐2 transmission among healthcare workers.^[^ ^72^ ^]^ Over 9 weeks during the pandemic, 231 participants were randomly assigned to either an “experimental” group instructed to use the AgNP solution, or a “control” group instructed to use conventional methods. The incidence of SARS‐CoV‐2 infection was markedly lower in the experimental group (1.8%) compared to the control group (28.2%), demonstrating an efficacy of 84.8%. These findings suggest that mouthwash and nasal rinse containing AgNPs may serve as effective measures in preventing SARS‐CoV‐2 transmission among healthcare workers exposed to COVID‐19 patients.^[^ ^72^ ^]^ Wieler et al. conducted a clinical trial to investigate the antiviral effects of AgNPs in patients with moderately severe to severe COVID‐19 pneumonia.^[^ ^73^ ^]^ The study was conducted in India from 2021 to 2022 and included 40 elderly patients (mean age 69.5 ± 13.5 years) diagnosed with severe COVID‐19 pneumonia, predominantly male (75.0%). This IIS was the first to explore intravenous administration of AgNPs as an adjuvant treatment alongside standard care. The AgNPs used were highly pure (99.99%), with a concentration of 1000 ppm and a size distribution of 10 nm, spherical in shape and soluble in water. Each patient received a total of 5.4 mg of AgNPs, equivalent to 30% of the human equivalent dose (HED). The results revealed that at day 5, the survival rate was significantly higher in the AgNPs treatment group (65.0% survival) compared to the control group receiving only standard COVID‐19 treatment (25.0% survival) (*p* < 0.05). This trend persisted at 30 days, with survival of 63.2% in the AgNPs group compared to 15.8% in the control group (*p* < 0.05). Furthermore, the AgNP‐treated group showed significant improvements in the need and duration of supplemental oxygenation compared to the control group (*p* < 0.05). In particular, no adverse events related to the administration of AgNPs were reported during the observation period.^[^ ^73^ ^]^ Gupta et al. conducted a study to assess the antiviral properties of silver nanoparticles (AgNPs) with varying surface characteristics, including bare, citrate‐stabilized, polyvinylpyrrolidone (PVP), and polyethylene glycol (PEG)‐modified AgNPs.^[^ ^74^ ^]^ Biocompatibility was evaluated using primary nasal epithelial cells and a human lung epithelial cell line. Two distinct pseudovirus neutralization assays were employed to investigate the ability of AgNPs to inhibit viral infection. Results indicated that unmodified nanoparticles exhibited greater efficacy against SAR‐CoV‐2 infection (EC_50_ ≈8.0 µg mL^−1^). Conversely, AgNPs modified with PVP or PEG failed to effectively block viral infection in the experimental model. Furthermore, the study found that AgNPs demonstrated non‐cytotoxic effects when exposed for 24 h at concentrations below 10 µg mL^−1^. These findings provide valuable insights into the potential of AgNPs as antiviral agents against SARS‐CoV‐2, highlighting the importance of surface modifications in their effectiveness.^[^ ^74^ ^]^ In the study conducted by Stanisic et al., high‐resolution magic‐angle spinning nuclear magnetic resonance spectroscopy (HR‐MAS NMR) was employed to assess the antiviral efficacy and biocompatibility of silver nanoparticles on coronavirus‐infected cells in vitro. Hesperetin (HST), a flavonoid, was utilized in the synthesis of silver nanoparticles to facilitate silver reduction and stabilize the resulting product.^[^ ^75^ ^]^ The synthesized biogenic silver nanoparticles exhibited an average diameter of ≈20 nm and were utilized to develop two distinct types of antiviral materials: colloids and 3D flexible nanostructured composites. Both materials demonstrated robust virucidal activity against coronaviruses in vitro models of cell infection, effectively inhibiting viral replication within cells while exhibiting minimal toxicity. These findings underscore the potential of silver nanoparticles, synthesized with HST, as promising candidates for combating coronavirus infections, offering both antiviral efficacy and biocompatibility.^[^ ^75^ ^]^ In the face mask industry, melt‐blown polypropylene fibers are widely utilized. Medvedev et al. employed silver nanoparticles (AgNPs) to modify cast brown polypropylene ribbon using a chemical metallization approach.^[^ ^76^ ^]^ The size of the silver crystallites ranged from 4 to 14, contingent upon the silver concentration. An in vitro study assessing the antiviral activity of the samples was conducted using VeroE6 cells. VeroE6 cell culture viability and typical cytopathic effects (CPE) were evaluated using an inverted microscope (Micromed) and an MTT assay. The antiviral efficacy of silver‐modified polypropylene tape was found to be correlated with the surface concentration of silver and the size of crystallites. Particularly noteworthy was the sample containing 1.0 g m^−2^ of silver, which demonstrated the highest antiviral activity, completely inhibiting viral replication.^[^ ^76^ ^]^ In their study, Morozova et al. aimed to identify the molecular targets of SARS‐CoV‐2, in particular the genomic RNA and the virion structural proteins S and N, using silver‐containing nanomaterials. To achieve this, they employed recombinant S2 and N proteins and viral RNA isolated from patient blood samples.^[^ ^77^ ^]^ Fluorescent Ag nanoclusters (NCs) smaller than 2 nm, citrate‐coated Ag nanoparticles (NPs) ranging from 20 to 120 nm in diameter, and nanoconjugates measuring 50–150 nm, composed of AgNPs with various protein shells, were synthesized using AgNO₃ and subsequently analyzed using TEM, AFM, UV‐Vis spectroscopy, and fluorescence spectroscopy. The artificial RNase complex, Li+[Ag+2Cys^2−^(OH^−^)_2_(NH_3_)_2_] (Ag‐2S), completely degraded SARS‐CoV‐2 RNA isolated from the blood samples of COVID‐19 patients, while other silver‐containing materials resulted in only partial RNA degradation. Additionally, treatment of the SARS‐CoV‐2 S2 and N recombinant antigens with AgNPs effectively inhibited their binding to specific polyclonal antibodies, as demonstrated by ELISA.^[^ ^77^ ^]^ Al‐Sanea et al. explored an alternative approach for inhibiting SARS‐CoV‐2, utilizing methanolic extracts from strawberry (Fragaria ananassa Duch.) and ginger (Zingiber officinale) for the synthesis of silver nanoparticles (AgNPs).^[^ ^78^ ^]^ The resulting AgNPs exhibited a spherical shape, with mean sizes of 5.89 nm and 5.77 nm for strawberry and ginger, respectively. To assess their antiviral potential against SARS‐CoV‐2, both the nanoparticles and extracts underwent testing using an MTT assay. Additionally, an in silico study was conducted to explore potential chemical compounds with anti‐SARS‐CoV‐2 properties, employing AutoDock Vina for molecular dynamic analysis. The findings revealed that the strawberry extract and green‐synthesized AgNPs from ginger exhibited the most potent antiviral activity. Furthermore, the docking study provided insights into the diverse interaction patterns between the compounds of strawberry and ginger with seven SARS‐CoV‐2 protein targets, including five viral proteins (Mpro, ADP ribose phosphatase, NSP14, NSP16, PLpro) and two human proteins (AAK1, Cathepsin L)^[^ ^78^ ^]^ (**Table** **2**).

**Table 2:** Characteristics and activity of AgNPs against SARS‐CoV‐2.

Type of nanoparticle	Synthesis method	Active concentrations	Refs.
Cotton fabric treated with AgNPs	Cotton cloth was cut into 2 × 2 cm^2^ pieces, cleaned with ultrasonic waves in acetone and distilled water, and dried. It was then immersed in a 1 mm AgNO₃ solution and exposed to UV light (254 nm) for 30 min. After drying at 50 °C, the cloth changed color from white to dark brown.	–	[61]
Face masks coated with silver nanoclusters	The radio frequency co‐sputtering process was conducted in a pure argon atmosphere, using 200 W RF power on a silica target and 3 or 5 W DC power on a silver target. This method produced coatings with varying amounts of silver nanoclusters, specifically lower amounts at 3 W (Ag3 W) and higher amounts at 5 W (Ag5 W).	–	[62]
AgNPs attachment to Polymeric Fibers	A solution of 1.0 mm silver nitrate and 1.0 mm tribasic sodium citrate was mixed, and 1.2 mm sodium borohydride was added to produce negatively charged AgNPs. The nanoparticles were washed by centrifugation and redispersed in Milli‐Q water at the desired concentration.	AgNPs at a concentration of 5 mg mL^−1^, along with polyethyleneimine (PEI) at 10 mg mL^−1^	[64]
AgNPs	AgNPs were acquired from Sigma Company	From 1 to 10 ppm	[65]
Surface coatings with citrate, polyvinylpyrrolidone (PVP), and branched polyethyleneimine (BPEI)	AgNPs were purchased from nanoComposix (San Diego, CA, USA)	From 1 to 10 ppm	[66]
Antiviral fabrics impregnated with AgNPs	A silver nitrate solution was prepared and the tissue was immersed in it at 60 °C. A filtered alcoholic extract of Eucalyptus globulus was gradually introduced and sodium hydroxide was added to raise the pH to 10. After 20 minutes of agitation, the tissue was removed, allowed to drain, and then dried in an oven at 100 °C for 15 min.	2.41 ppm	[68]
Polyurethane (PU)/AgNP	AgNPs were acquired by Merck	–	[69]
Ceramic‐coated AgNPs into thermoplastic polyurethane (TPU) plates	AgNPs were acquired from Esenttia	<100 µg mL^−1^	[70]
PVC/AgNP	AgNPs were purchased from TechNano Solutions	0.3 wt% AgNPs	[71]
Mouthwash and nasal rinse containing AgNP	ARGOVIT AgNPs, produced by the Investigation and Production Center Vector‐Vita Ltd., were used.	From 0.5% to 0.0004% of metallic silver	[72]
AgNPs polyvinylpyrrolidone (PVP), and polyethylene glycol (PEG)‐modified	AgNPs were acquired from Sigma	Concentrations below 10 µg mL^−1^	[74]
AgNPs with the flavonoid Hesperetin (HST)	AgNPs were synthesized by dissolving hesperetin in sodium hydroxide, adding silver nitrate in a 1:1 ratio, and adjusting the pH to 7.4 using diluted hydrochloric acid.	–	[75]
AgNPs to modify cast brown polypropylene ribbon	Metallic silver was coated onto the fiber surface through a chemical metallization process, involving treating the polymer material with tin chloride solution for sensitization, silver nitrate solution for activation, and a solution of silver ammonia complexes for metallization.	1.0 g m^−2^	[76]
AgNPs	Ginger and Strawberry AgNPs were synthesized by dissolving 0.05 g of methanolic extract in 3 mL DMSO, then the addition of 0.5 mL of DMSO extract and 1 mL 1 m NaOH to 10 mL 1 mm AgNO_3_. The mixture was kept in a water bath for 10 min at 60 °C.	0034 µg mL^−1^ and 0,0062 µg mL^−1^	[78]

### 2.3 Herpes Simplex Virus (HSV)

**Figure 3:**
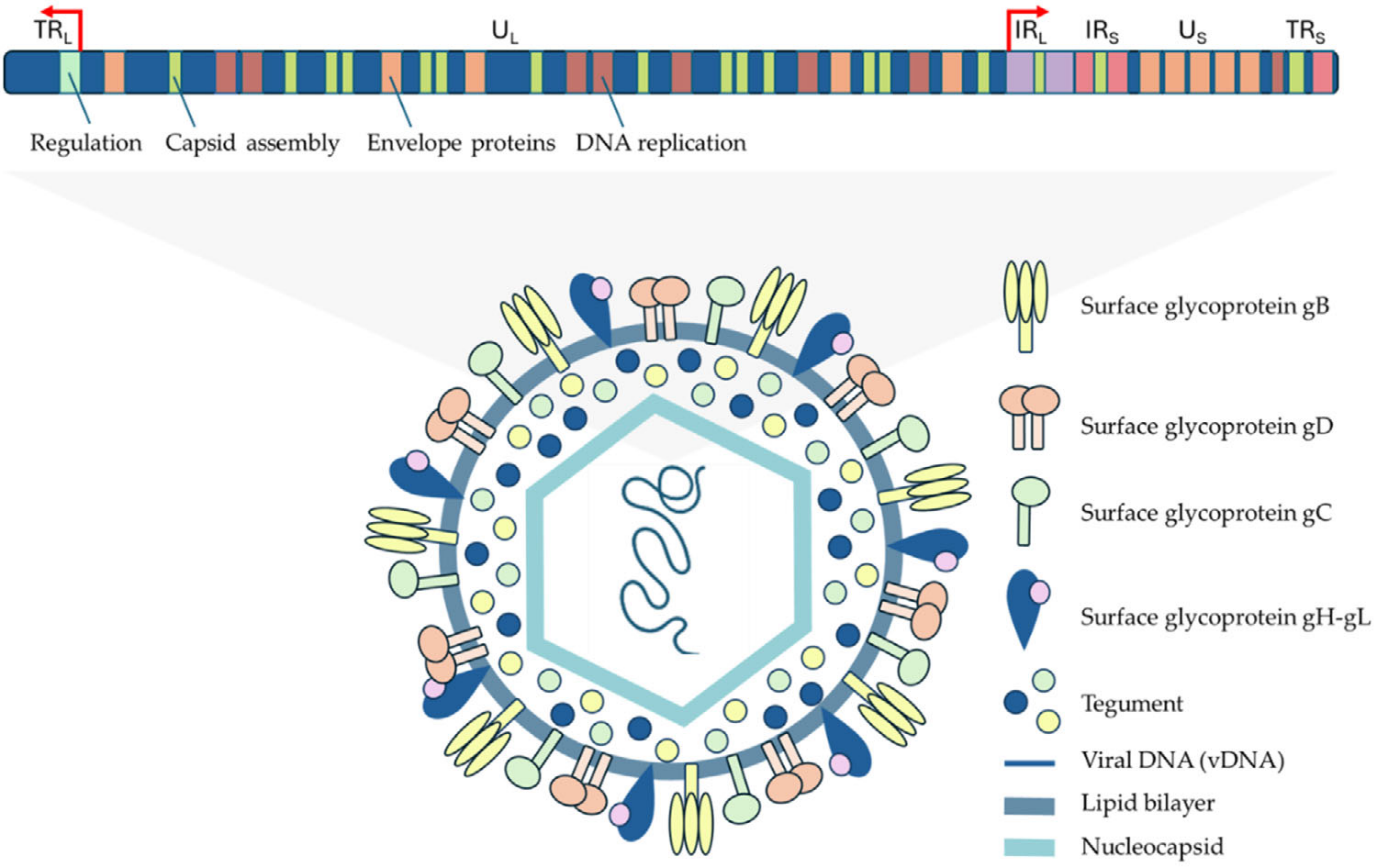
HSV virion structure and genome organization.

The family of alphaherpesviruses includes the widespread human pathogens known as herpes simplex viruses, HSV‐1 and HSV‐2. They are genetically complex double‐stranded DNA viruses that have adapted to replicate in human neurons and epithelial cells. Viral gene expression, DNA replication, capsid assembly and genome packaging occur in the infected cell's nucleus. Mature nucleocapsids leave the nucleus by envelopment in the inner nuclear membrane to localize within cytoplasmic organelles.^[^ ^79^ ^]^ This process culminates in the formation of mature infectious particles, which are sorted into sensory neuron terminals or epithelial cell junctions (**Figure** **3**). HSV is responsible for a spectrum of conditions including herpes labialis, pharyngitis, herpetic gingivostomatitis, keratitis, encephalitis, and neonatal herpes infection. After initial infection, the virus establishes a lifelong latent presence within sensory and autonomic neural ganglia. HSV poses serious risks, potentially leading to fatal infections in newborns and individuals with compromised immune systems, along with physical impairments, social ostracization, and psychological distress. Although uncommon, mutations in the viral DNA polymerase gene can lead to antiviral resistance.^[^ ^79^ ^]^ However, it's more common for mutations in the viral thymidine kinase gene to confer resistance to drugs like acyclovir.^[^ ^80^ ^]^ The emergence of HSV resistance to acyclovir presents a significant clinical challenge, particularly for patients with weakened immune systems undergoing prolonged treatment. Rarely, resistant isolates can lead to visceral dissemination and severe, progressive, and incapacitating mucosal disease. The emergence of resistance to drugs has prompted the quest for alternative modalities of treatment with distinct mechanisms of action. Silver nanoparticles show potential in this direction. Depending on their mechanism of action, AgNP silver nanoparticles can exert antiviral effects by inhibiting viral adhesion to host cells, preventing viral entry into cells, or degrading viral DNA. Multiple research groups have developed AgNPs aiming to use their antiviral properties against HSV. Fayaz et al. engineered a polyurethane condom incorporating silver nanoparticles (Ag‐NPs), denoted as PUC, and assessed its efficacy against HIV‐1 and HSV‐2.^[^ ^81^ ^]^ The nanoparticles were synthesized chemically, exhibiting a size distribution ranging from 30 to 60 nm. Their integration with the device was facilitated by interacting with the nitrogen atom within the polyurethane condom matrix. The evaluation involved examining the impact of condom interaction with HeLa cells, 293 T cells, and C8166 cells. Remarkably, the Ag‐NP‐coated PUC showed negligible effects on cell viability and proliferation following a 3‐h exposure period. Furthermore, inhibition assays against HSV‐1 and HSV‐2 were conducted utilizing Vero cells. Upon exposure to the Ag‐NP‐coated PUC, both viral strains exhibited complete loss of infectivity. This suggests a potent antiviral effect conferred by the nanoparticle‐coated condom. Moreover, the risk of chronic toxicity is mitigated by the robust binding of bioactive AgNPs to the condom material, enhancing its safety profile.^[^ ^81^ ^]^ Hu et al. endeavored to elucidate the inhibitory mechanisms of silver nanoparticles (Ag‐NPs) against HSV‐2.^[^ ^82^ ^]^ The nanoparticles utilized were commercially acquired Nano Silver‐PVP, comprising 0.2 wt% polyvinylpyrrolidone (PVP) and possessing a particle size of 30–40 nm. Cytotoxicity was assessed via the MTT assay on Vero cells, while the inhibitory efficacy of Ag‐NPs against HSV‐2 was evaluated through plaque assays. Remarkably, Ag‐NPs at a concentration of 100 µg mL^−1^ exhibited negligible toxicity towards Vero cells. In viral pretreatment assays, these nanoparticles significantly attenuated viral progeny production. Moreover, in viral post‐treatment assays at the same concentration, Ag‐NPs impeded the viral replication phase. Notably, administration of Ag‐NPs prior to or promptly after initial viral exposure, at non‐cytotoxic concentrations, effectively suppressed HSV‐2 replication and induced alterations in viral structure.^[^ ^82^ ^]^ Morone et al. developed a novel method to synthesize ligand‐free silver nanoparticles (PLAL‐AgNPs) using liquid pulsed laser ablation. The resulting nanoparticles had a spherical shape with an average diameter of 82.5 nm. These AgNPs demonstrated minimal cytotoxicity on Vero‐76 cells while exhibiting strong antiviral activity against HSV‐1. Notably, only 1.6 × 10⁶ AgNPs were sufficient to disrupt the viral envelope, effectively inhibiting the infection process.^[^ ^83^ ^]^ Tannic acid‐modified silver nanoparticles (AgNPs) have gained widespread recognition as a microbicide due to their adjunctive properties in treating genital herpes infections and augmenting both cellular and humoral anti‐HSV‐2 responses. Orlowski et al. conducted a comprehensive investigation into the antiviral efficacy of tannic acid modified AgNPs both in vivo using female C57BL/6 mice and in vitro with African green monkey kidney cells.^[^ ^84^ ^]^ In the in vivo study, mice were intravaginally inoculated with 10^4^ PFU/mouse of HSV‐2 strain 333, which had been pre‐incubated for 1 h with or without 5 mg mL^−1^ AgNPs. To assess the antiviral activity of AgNPs in infected animals, mice were irrigated three times with 100 ml of 5 mg mL^−1^ 33 nm AgNPs at either 3‐ or 18 h post‐infection (p.i.). At the peak of local vaginal infection (48 h p.i.), incubation of HSV‐2 with all tested AgNPs (13, 33, and 46 nm) led to a significant reduction in viral titers as measured by PCR. Interestingly, both the 13 and 46 nm AgNPs exhibited a comparable antiviral effect during HSV‐2 infection in vivo, whereas the 33 nm AgNPs demonstrated the highest efficacy. This underscores the size‐dependent nature of the antiviral activity of tannic acid‐modified AgNPs, which necessitates specific interactions to impede virus attachment, replication, and subsequent transmission.^[^ ^84^ ^]^ Szymanska et al. also explored the application of tannic acid‐modified silver AgNPs, employing a distinct approach for the effective management of herpes virus infections through the development of a mucoadhesive gel formulation system.^[^ ^85^ ^]^ The antiviral activity against HSV‐1 and HSV‐2 was evaluated both in vitro using an immortal human keratinocyte cell line and in vivo utilizing a mouse model of genital HSV‐2 infection. The semi‐solid hydrogel formulation demonstrated a notable advantage by prolonging the exposure period of AgNPs at the site of infection while concurrently impeding viral invasion and cell‐to‐cell transmission. This innovative strategy offers promising prospects for combating herpes virus infections by enhancing the retention and efficacy of tannic acid‐modified AgNPs through a mucoadhesive gel formulation.^[^ ^85^ ^]^ El‐Mohamady et al. investigated the inhibitory effects of Ag‐NPs on the viral replication of bovine herpesvirus‐1 (BoHV‐1), serving as a model system. The Ag‐NPs were synthesized chemically, yielding particles ranging in size from 15 to 50 nm.^[^ ^86^ ^]^ Cytotoxicity was assessed on MDBK cells via the MTT assay, while the cytopathic effects (CPE) of BoHV‐1 and the protective effects of Ag‐NPs against infection were tracked using neutral red (NR) staining. The findings indicated that AgNPs at a concentration of 24 µg mL^−1^ exhibited no adverse effects on MDBK cells. Notably, at this safe concentration, Ag‐NPs effectively shielded MDBK cell cultures from BoHV‐1 infection when a mixture of Ag‐NPs and BoHV‐1 suspension was incubated prior to cell infection. This underscores the potential of Ag‐NPs as a protective agent against BoHV‐1 infection in cellular cultures, highlighting their promise for further exploration in antiviral strategies.^[^ ^86^ ^]^ In a study conducted by Krzyzowska et al., chemically synthesized AgNPs were modified with lactoferrin (LF‐Ag/AuNP) to achieve a size of 10 nm.^[^ ^87^ ^]^ Lactoferrin, recognized for its potent suppression of human herpes virus types 1 and 2, also exhibits immunomodulatory properties by stimulating the production of various cytokines in the genital tract. The antiviral efficacy of these modified nanoparticles was evaluated through both in vitro and in vivo experiments targeting HSV‐2. LF‐AgNPs were assessed using HaCaT fibroblasts and vaginal VK‐2‐E6/E7 keratinocytes to measure cell viability via the MTT assay. HSV‐2 was cultured with Vero cells, and viral titers were determined either through plaque assay or quantitative PCR (qPCR). In murine HSV‐2 vaginal infection models, viral titers upon treatment were examined. Phenotypic analysis of vaginal tissue was conducted using flow cytometry, and response identification was facilitated by PCR. The analysis revealed that lactoferrin‐functionalized AgNPs effectively prevented HSV‐2 infection by inhibiting virus attachment, penetration, and subsequent infection when employed in pretreatment. Moreover, infected mice treated with LF‐Ag/AuNPs exhibited lower viral titers in both vaginal tissues and the spinal cord. These findings underscore the potential of lactoferrin‐modified AgNPs as a promising therapeutic approach for combating HSV‐2 infection.^[^ ^87^ ^]^ Subsequently, Krzyzowska et al. evaluated the potential of epigallocatechin gallate (EGCG)‐modified 30 nm AgNPs (EGCG‐AgNPs) against both HSV‐1 and HSV‐2.^[^ ^88^ ^]^ The antiviral efficacy and toxicity of EGCG‐AgNPs were examined in human HaCaT and VK‐2‐E6/E7 keratinocytes. The standard plaque formation assay (PFU mL^−1^) was conducted using Vero cell cultures. Furthermore, the impact of EGCG‐AgNPs on HSV‐1 and HSV‐2 infection and immune responses was evaluated in mouse models of intranasal HSV‐1 and genital HSV‐2 infections. Male and female C57BL/6 mice, aged 6 to 8 weeks, were used for in vivo experiments. The results revealed that the functionalized nanoparticles exhibited superior inhibition of HSV attachment and entry compared to EGCG alone. Furthermore, infected mice treated with EGCG‐AgNP demonstrated reduced viral titers and increased immune responses. This included increased infiltration of dendritic cells, monocytes, CD8+ T cells, NK cells, and elevated levels of interferon‐γ and interleukin‐12. These findings suggest the potential of EGCG‐AgNPs as a promising therapeutic strategy against HSV infections, warranting further investigation into their clinical applicability and safety profile.^[^ ^88^ ^]^ Several studies investigating the impact of silver nanoparticles (AgNPs) on herpes simplex viruses have utilized materials derived from environmentally friendly processes. Gaikwad et al. explored the antiviral potential of AgNPs synthesized via mycosynthesis against both HSV‐1 and HSV‐2.^[^ ^89^ ^]^ The mycosynthesized AgNPs were derived from the fungal cell filtrates of five distinct species, namely Alternaria, *F. oxysporum*, Curvularia, *C. indicum*, and Phoma. The resulting nanoparticles exhibited sizes ranging from 7–20 nm for Alternaria, 4–13 nm for *F. oxysporum*, 5–23 nm for Curvularia, 10–31 nm for *C. indicum*, and 7–20 nm for Phoma species. Cytotoxicity assessments were performed on Vero cells across various concentrations (1, 5, 10, 50, and 100 µg mL^−1^) at different time intervals. Subsequently, concentrations ranging from 0.5 to 5 µg mL^−1^ were selected for further experimentation based on these results. The findings indicated robust inhibition of replication efficiency for HSV‐1, while HSV‐2 exhibited minor susceptibility to the AgNPs. Notably, AgNPs biosynthesized with *F. oxysporum* and *Curvularia* species, exhibiting sizes of 4–13 nm and 5–23 nm, respectively, demonstrated potent antiviral activity (80–90% inhibition) against HSV‐1 with minimal cytotoxicity to Vero cells. In contrast, AgNPs derived from Alternaria and Phoma species, with sizes ranging from 7 to 20 nm, exhibited comparatively weaker antiviral activity. Thus, the study suggests that AgNPs of smaller sizes tend to yield more favorable outcomes in inhibiting virus replication.^[^ ^89^ ^]^ Zeedan et al. conducted an in vivo study exploring green nanotechnology by employing aqueous extracts of olive leaves and natural honey for the synthesis of silver nanoparticles (AgNPs).^[^ ^90^ ^]^ The potential antiviral activities of these nanoparticles against bovine herpesvirus‐1 (BoHV‐1) were evaluated both in vitro and in vivo. The formation of spherical and prismatic nanoparticles ranging from 10 to 50 nm in size was observed. To assess cytotoxicity, an MDBK cell culture was employed alongside a modified enzyme‐linked immunosorbent assay (ELISA) and cytopathic effects (CPE) evaluation for detecting cytopathogenic changes. The tested nanoparticles demonstrated safety for MDBK cells at concentrations ranging from 24 µg mL^−1^ to 50 µg mL^−1^ and effectively prevented BoHV‐1 infection in the MDBK cell culture at a nontoxic concentration of 25 µg mL^−1^. The selectivity index (SI), represented by the CC50/TCID50 ratio, highlighted the most beneficial index at concentrations of 25 µg mL^−1^ and 50 µg mL^−1^, with reductions in BoHV‐1 titers from 7 to 2.5 and 1.5, respectively. Furthermore, the green nanoparticles were deemed safe for experimental animals receiving AgNPs at dosages of 50 µg k^−1^g/day. However, molecular detection using PCR revealed the presence of BoHV‐1 in nasal swabs of positive control rabbits within the first 2 days after infection, although the virus was undetectable thereafter.^[^ ^90^ ^]^ The wide diversity of brown algae within the Sargassum genus presents a largely untapped reservoir of biological activity. Dhanasezhian et al. explored the synthesis of silver nanoparticles (AgNPs) derived from *Sargassum wightii* (Sw‐Ag) and evaluated their potential as anti‐HSV agents.^[^ ^91^ ^]^ Their cytotoxicity study on Vero cells revealed that Sw‐Ag exhibited toxicity at doses of 5 and 10 µL, while lower doses (1 and 2.5 µL) demonstrated non‐toxic effects, producing cell viability of 97% respectively and 84.58%. The antiviral efficacy of Sw‐Ag nanoparticles was evaluated by measuring the reduction of virus‐induced cytopathic effects (CPE). Sw‐Ag nanoparticles, administered at volumes of 0.5, 1, 2.5, and 5 µL, effectively mitigated HSV‐induced CPEs in a dose‐dependent manner. Notably, treatment with 1 µL of Sw‐Ag nanoparticles resulted in a 70% reduction in CPE for both HSV‐1 and HSV‐2. Furthermore, approximately 2.5 µL of Sw‐Ag nanoparticles reduced CPE by 70% for both viral strains. In contrast, Sw‐Au nanoparticles demonstrated less reduction in CPE (70%) for HSV‐1 and HSV‐2 at volumes of 10 and 25 µL, respectively. Although Sw‐Ag nanoparticles at 5 µL showed stronger antiviral activity, they induced morphological changes in Vero cells due to their toxicity.^[^ ^91^ ^]^ Haggag et al. utilized aqueous and hexane extracts derived from *Lampranthus coccineus* and *Malephora lutea* to synthesize AgNPs and investigated their antiviral efficacy against various viruses, including HSV‐1, employing the MTT assay.^[^ ^92^ ^]^ Metabolomics profiling was performed using UPLC‐MS, and molecular docking was executed via Autodock4. The synthesized nanoparticles derived from the aqueous and hexane extracts of *Lampranthus coccineus* exhibited a spherical morphology with average diameters ranging from 10.12 to 27.89 nm, while those from *Malephora lutea* ranged from 8.91 to 14.48 nm. Notably, AgNPs produced from the hexane extract of *Malephora lutea* demonstrated potent antiviral activity, with IC50 values against the HSV‐1 virus recorded at 36.36 µg mL^−1^. Conversely, AgNPs derived from the hexane extract of *Malephora lutea* did not display significant antiviral efficacy against HSV‐1. Aqueous extract‐derived samples showed negligible effectiveness. The observed antiviral properties of the nanoparticles are likely attributed to their interaction with viral envelope glycoproteins, thereby impeding viral entry into host cells. Additionally, these nanoparticles may infiltrate viral cells and modulate antiviral activity through interactions with viral genomic materials.^[^ ^92^ ^]^ Ramadan et al. assessed the antiviral effectiveness of AgNPs synthesized from the aqueous extract of *Melaleuca alternifolia* leaves against HSV‐1 and HSV‐2. The AgNPs demonstrated robust antiviral activity, eliciting a 44.0% reduction in cytopathic effect against HSV‐1 and a 45.04% reduction against HSV‐2.^[^ ^93^ ^]^ Hasanin et al. synthesized eco‐friendly silver nanoparticles (AgNPs) from bacterial isolate extracts and integrated them into a tertiary nanocomposite comprising starch, oxidized cellulose, and ethyl cellulose (Ag‐NC).^[^ ^94^ ^]^ The antimicrobial and antiviral properties of the resulting nanocomposite were investigated, particularly against HSV‐1, utilizing the MTT assay with Vero cells. Cytopathogenic effects (CPE) or morphological alterations in cells were documented using an inverted microscope. Pre‐treatment of viruses with Ag‐NPs (6.25 µg mL^−1^) or Ag‐NC (12.5 µg mL^−1^) for 1 h prior to infection resulted in the absence of significant CPE compared to untreated control cells. Incubation of the virus with Ag‐NPs (1.56, 3.12, 6.25 µg mL^−1^) or Ag‐NPs nanocomposite (3.12, 6.25, 12.5 µg mL^−1^) for one hour with cells demonstrated a notable dose‐dependent decrease in viral replication. Notably, the concentration of 6.25 µg mL^−1^ Ag‐NPs exhibited the most substantial efficacy against all tested viruses (HSV‐1, Adenovirus, and CoxB2), with a reduction of 61.7% ± 6.6% observed in the case of HSV‐1. Similarly, the same concentration (6.25 µg mL^−1^) of Ag‐NC displayed moderate antiviral activity in cultured cells.^[^ ^94^ ^]^ AgNPs produced using a biogenic approach by Srisrimal et al., already discussed previously regarding the H1N1 virus, were also tested for their virucidal efficacy against HSV‐1. While AgNPs had 50% cytotoxicity in Vero cells at a concentration of 197 µg mL^−1^, and the 50% inhibitory concentration (IC50) against HSV‐1 was only achieved at 19.6 µg mL^−1^. AgNPs were found to be effective in a dose‐dependent manner.^[^ ^51^ ^]^ El‐Sheekh et al. employed strains of blue‐green algae, namely *Oscillatoria* sp. and *Spirulina platensis*, for the green synthesis of Ag2O/AgO‐NPs and Au‐NPs.^[^ ^95^ ^]^ The Ag_2_O/AgO‐NPs obtained exhibited a spherical morphology with dimensions ranging from 14.42 to 48.97 nm, while the Au‐NPs manifested as octahedral, pentagonal, and triangular structures with dimensions ranging from 15.60 to 77.13 nm. The antiviral activity against HSV‐1 was assessed based on the reduction of cytopathic effects (CPE). Cytotoxicity was evaluated on Vero cells using the MTT assay. Significantly reduced HSV‐1 cytopathic activity was observed upon treatment. Specifically, a remarkable 90% reduction in HSV‐1 CPE was achieved by applying Ag_2_O/AgO‐NPs and Au‐NPs at 31.25 µL^[^ ^96^ ^]^ (**Table** **3**).

**Table 3:** Characteristics and Activity of AgNPs Against HSV.

Type of nanoparticle	Synthesis method	Viruses	Active concentrations	Refs.
Incorporation of AgNPs in polyurethane condom (PUC)	For the synthesis of Ag‐NPs, a 7.0 mm trisodium citrate solution was boiled and 1.0 mL of 0.1 M silver nitrate was added to start the reaction. The color change of the solution from colorless to yellow signaled AgNPs formation.	HSV‐1, HSV‐2	–	[81]
Nano Silver‐PVP with 0.2 wt% polyvinylpyrrolidone (PVP)	AgNPs was purchased from Shenzhen Baiwang Biological Science and Technology Ltd	HSV‐2	100 µg mL^−1^	[82]
Tannic acid‐modified AgNPs	AgNPs of varying sizes were synthesized by adjusting the concentrations and conditions of the reagents. The process involved adding sodium citrate, tannic acid, and sodium borohydride to a silver nitrate solution, leading to a color change in the final solution that indicated AgNPs formation. The pH values differed for each synthesis, correlating with the size of the AgNPs produced.	HSV‐2	5 mg mL^−1^	[84]
Tannic acid‐modified AgNPs through a mucoadhesive gel formulation system (GRAS) as a semi‐ solid hydrogel formulation	A sterile aqueous solution of silver nitrate (0.017%, 95.2 g) served as the metal salt precursor, while sodium citrate (4%, 4.2 g) acted as the reducing agent and tannic acid (5%, 0.6 g) functioned as both a reducing and stabilizing agent.	HSV‐1, HSV‐2	25‐50 ppm	[85]
Ag‐NPs	AgNPs were synthesized through the chemical reduction method by adding sodium citrate, PVP, and H_2_O_2_ to a freshly prepared AgNO_3_ solution while stirring vigorously. After a few minutes, NaBH_4_ was added to create colloidal AgNPs. The mixture was stirred for 3 h and then stored in the dark at 4 °C until use.	BoHV‐1	24 µg mL^−1^	[86]
AgNPs with lactoferrin	AgNPs were synthesized by reducing silver nitrate with sodium borohydride in the presence of sodium citrate and tannic acid.	HSV‐2	–	[87]
Epigallocatechin gallate (EGCG)‐modified AgNPs	Sodium citrate (3 g, 1%) was refluxed with deionized water (75 mL) for 15 minutes. Silver nitrate (2.6 mL, 1%) and silver seeds (10 g) were then added, and the mixture was refluxed for an hour. A second portion of sodium citrate and silver nitrate was added, followed by another hour of reflux. The resulting AgNPs had a concentration of 355 ppm, which was subsequently diluted to 100 ppm for further use.	HSV‐1, HSV‐2	7,5 µg mL^−1^	[88]
AgNPs	Fungi were cultured in potato dextrose broth, filtered, and washed to remove residues. The biomass was suspended in distilled water for 48 h, and the resulting cell filtrate was treated with silver nitrate at a concentration of 1 mm.	HSV‐1, HSV‐2	From 100 to 1 µg mL^−1^, then from 0.5 to 5 µg mL^−1^	[89]
AgNPs	AgNPs were synthesized using local natural honey from Egypt by dissolving two concentrations of honey (30 g and 40 g) in deionized water (80 mL and 70 mL, respectively). From each honey solution, a 15 mL aliquot was mixed with 20 mL of 1 mm AgNO3 and stirred for 1 minute at 60 °C. The pH was then adjusted to 8.55, 9, 9.5, and 10.5 using NaOH to facilitate the reduction of silver ions.	BoHV‐1	From 25 to 50 µg mL^−1^	[90]
AgNPs	Algal samples were washed, shade‐dried, and ground into a fine powder. To synthesize AgNPs, 1 g of the seaweed powder was soaked in 100 mL of double‐distilled water for 24 h, then filtered. The filtrate (10 mL) was combined with 90 mL of a 10⁻^3^ M silver nitrate solution and stirred for 15 h, leading to a dark brown color that indicated AgNP formation.	HSV‐1, HSV‐2	From 1 to 2.5 µL	[91]
AgNPs	AgNPs were synthesized by macerating 10 g of air‐dried aerial parts of *L. coccineus* and *M. lutea* in 100 mL of distilled water at 60 °C for 30 minutes. The extract was filtered through Whatman No. 1 filter paper, and then mixed with 1 mm silver nitrate in a 2:10 ratio and incubated at 60 °C for 10 minutes to facilitate AgNP biosynthesis.	HSV‐1	36.36 µg mL^−1^	[92]
AgNPs	For the AgNP synthesis, 2.0 mg mL^−1^ stock solutions were created from the aqueous extract of M. alternifolia leaves. A total of 90 mL of a 5.0 mm silver nitrate solution was mixed with 10 mL of the stock extract and left at room temperature for 24 h. The formation of AgNPs was initially indicated by a color change of solution	HSV‐1, HSV‐2.	0.02 mg mL^−1^	[93]
AgNPs in nano composition with starch, oxidized cellulose, and ethyl cellulose (Ag‐NC)	A cell‐free extract from an overnight‐cultured isolated Bacterial strains were used to synthesize silver nanoparticles (Ag‐NPs) from a 1 mm silver nitrate (AgNO3) precursor. The formation of Ag‐NPs was visually indicated by a browning reaction and confirmed with a UV‐visible spectrophotometer.	HSV‐1	6.25 µg mL^−1^	[94]
AgNPs	A cell‐free extract from an isolated bacterial strain grown overnight was used to synthesize Ag‐NPs from 1 mm silver nitrate precursor.	HSV‐1	19.6 µg mL^−1^	[51]

### 2.4 Human Immunodeficiency Virus (HIV)

**Figure 4:**
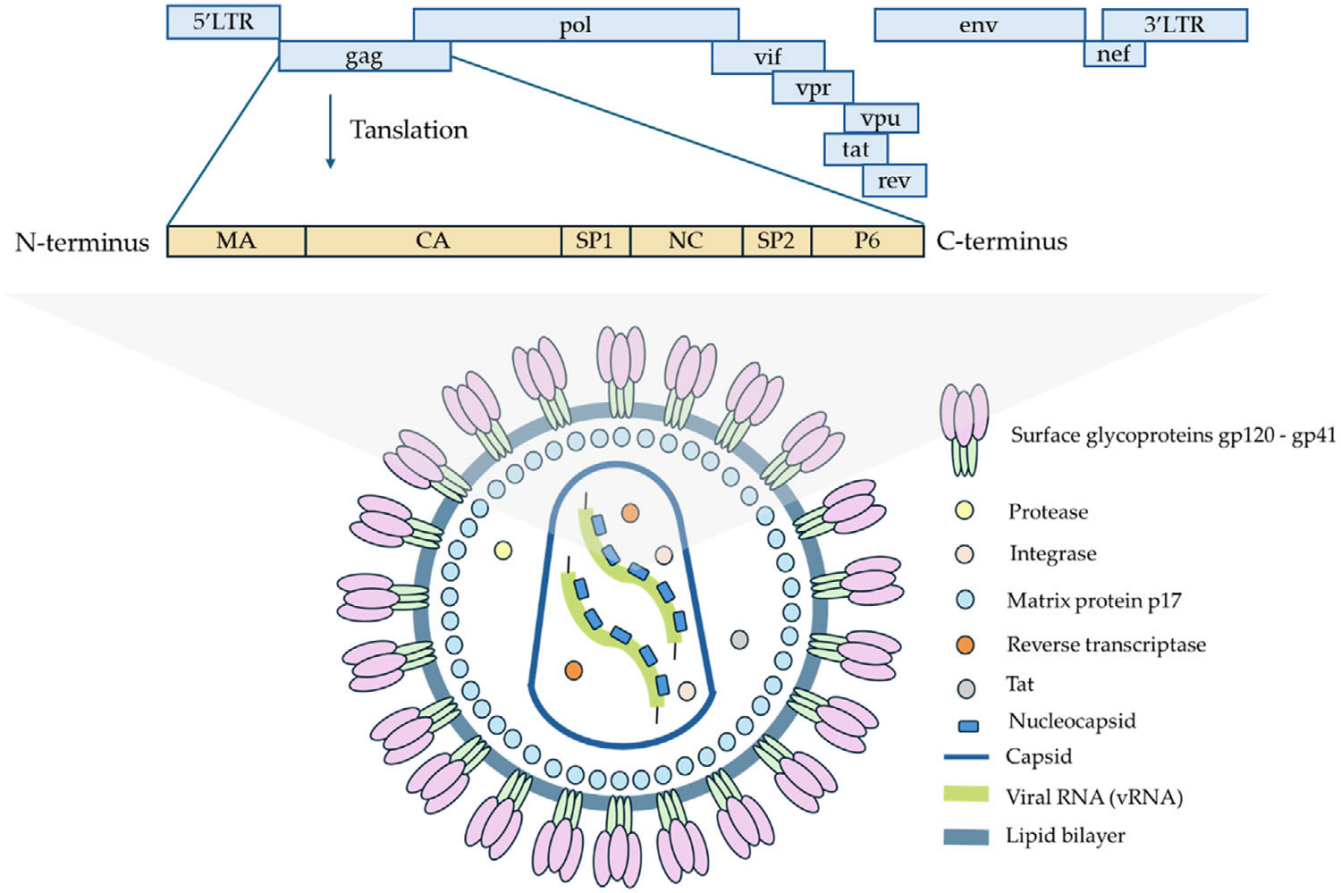
HIV virion structure and genome organization.

Human immunodeficiency virus (HIV) is the etiological agent responsible for inducing acquired immunodeficiency syndrome (AIDS) in humans. HIV belongs to the genus Lentivirus within the Retroviridae family. Two distinct variants of HIV have been characterized: HIV‐1 and HIV‐2. HIV‐1, initially identified as lymphadenopathy‐associated virus (LAV) and human T‐lymphotropic virus 3 (HTLV‐III), was the first to be isolated. It exhibits higher virulence and transmissibility compared to HIV‐2 and predominates in global HIV infections.^[^ ^97^ ^]^ The virion of HIV is ≈120 nm in diameter and exhibits a spherical morphology. Its genetic material, which encoding nine genes, is enclosed within a conical capsid composed of 2000 copies of the viral protein p24. The genomic content of HIV‐1 consists of two copies of positive‐sense, noncovalently linked, unspliced single‐stranded RNA molecules. These RNA strands do not function independently; rather, they form a tightly packed dimeric structure within the virion. Spanning a length of 9749 nucleotides, the RNA encompasses essential features including a 5′ cap (Gppp), a 3′ poly(A) tail, and numerous open reading frames (ORFs). Long ORFs within the viral genome encode structural proteins, whereas smaller ORFs govern pivotal aspects of the viral life cycle, including attachment, membrane fusion, replication, and assembly. Single‐stranded RNA is intricately associated with various viral components such as nucleocapsid proteins p7, the late assembly protein p6, as well as key enzymes vital for virion maturation, such as reverse transcriptase and integrase.^[^ ^98^ ^]^ This tight association ensures the coordinated orchestration of viral replication and assembly processes. An enveloping matrix, comprised primarily of the viral protein p17, surrounds the capsid, ensuring the structural integrity of the virion particle. Upon entry into the target cell, reverse transcriptase, an encoded viral enzyme within the particle, catalyzes the conversion of the viral RNA genome into double‐stranded DNA. Subsequently, the resultant viral DNA integrates into the host cellular DNA facilitated by the viral enzyme integrase along with host cofactors (**Figure** **4**).^[^ ^99^ ^]^ HIV poses a significant global health challenge due to its facile transmission through bodily fluids such as blood, genital secretions, and breast milk. Additionally, HIV, alongside other sexually transmitted infections (STIs), constitutes a formidable threat to healthcare systems worldwide. Highly active antiretroviral therapy (HAART), a combination of medications aimed at suppressing HIV replication, has markedly improved the overall health and longevity of millions of HIV‐infected individuals. However, the emergence of drug‐resistant mutations presents a persistent risk to treatment efficacy and hastens disease progression on a global scale. Moreover, conventional antiretroviral medications encounter limitations such as extensive first‐pass metabolism, gastrointestinal degradation, and issues related to solubility and stability. Consequently, researchers are actively exploring novel therapeutic approaches, including innovative drug delivery systems (NDDS) designed to enhance drug efficacy and patient outcomes.^[^ ^100^ ^]^ In recent investigations, the potential of AgNPs has been examined in this context, signaling a promising avenue for future research and therapeutic development. In 2005, Elechiguerra et al. conducted a study elucidating the interaction between silver nanoparticles and HIV‐1.^[^ ^21^ ^]^ The investigation involved nanoparticles of distinct surface compositions: foamy carbon (with a size distribution of 16.19 ± 8.68 nm), poly(N‐vinyl‐2‐pyrrolidone) (PVP) (with a size distribution of 6.53 ± 2.41 nm), and bovine serum albumin (BSA) nanoparticles (with an average size of 3.12 ± 2.00 nm). High‐angle annular dark field (HAADF) scanning transmission electron microscopy served as the primary technique to investigate the interaction between silver nanoparticles and HIV‐1. The efficacy of silver nanoparticles in inhibiting HIV‐1 infectivity was assessed through experimentation involving MT‐2 CD4^+^ cells and HIV‐1 cMAGI reporter cells. The cytotoxicity of each nanoparticle variant against MT‐2 cells was evaluated utilizing the Trypan Blue exclusion test. Results demonstrated that across all three nanoparticle formulations, at silver concentrations exceeding 25 µg mL^−1^, viral infectivity was significantly diminished to the extent that syncytium formation could not be detected. Moreover, in each nanoparticle formulation, a dose‐dependent reduction in HIV‐1 infectivity was observed, with an IC50 level where only mild cellular toxicity was observed. Furthermore, the investigation indicated that silver nanoparticles exhibit a size‐dependent binding affinity to the knobs of the HIV gp120 glycoprotein, particularly within the 1–10 nm size range. This binding mechanism effectively impedes the attachment of the virus to host cells, thereby preventing viral infection.^[^ ^21^ ^]^ To delve deeper into the mechanism underlying HIV‐1 inactivation by AgNPs, the same research group conducted a comprehensive study utilizing commercially available 30–50 nm silver nanoparticles coated with 0.2 wt% polyvinylpyrrolidone (PVP‐coated AgNPs). Various assays were employed, including virus adsorption assays, cell‐based fusion assays, gp120/CD4 capture ELISA, and virucidal activity assays with cell‐free virus. The study utilized HeLa‐CD4‐LTR‐b‐gal cells (expressing both CXCR4 and CCR5), MT‐2 cells (a lymphoid human cell line expressing CXCR4), and human peripheral blood mononuclear cells (PBMC) to assess cytotoxicity. A luciferase‐based assay was employed to determine the CC_50_ of AgNPs, yielding values of 3.9 ± 1.6 mg mL^−1^ against HeLa‐CD4‐LTR‐b‐gal cells, 1.11 ± 0.32 mg mL^−1^ against human PBMC, and 1.3 ± 0.58 mg mL^−1^ against MT‐2 cells. Results revealed that HIV‐1 infection, irrespective of tropism or resistance profile, was hindered by the AgNPs. This inhibition occurred through the binding of the nanoparticles to gp120, thereby impeding CD4‐dependent virion binding, fusion, and subsequent infection. PVP‐coated AgNPs demonstrated efficacy in blocking both cell‐free and cell‐associated HIV‐1 infection, exhibiting virucidal activity against the virus.^[^ ^101^ ^]^ In a subsequent study, Lara et al. investigated the efficacy of polyvinylpyrrolidone‐coated silver nanoparticles (PVP‐coated AgNPs) as a potential topical vaginal microbicide for inhibiting the spread of HIV‐1 infection. The study utilized human cervical cell cultures as an in vitro model to simulate in vivo conditions. When incorporated into a non‐spermicidal gel at a concentration of 0.15 mg mL^−1^, PVP‐coated AgNPs demonstrated significant efficacy in blocking both cell‐associated and cell‐free HIV‐1 transmission. Notably, these nanoparticles exhibited no toxicity to the cervical cell explant, even after continuous exposure for 48 h. Furthermore, a brief 1‐min pretreatment of the explant with PVP‐coated AgNPs was adequate to prevent HIV‐1 transmission. Moreover, a more extended 20‐minute pretreatment followed by thorough washing resulted in sustained prevention of transmission for up to 48 h in this model. These findings underscore the potential of PVP‐coated AgNPs as an effective and safe microbicide strategy against HIV‐1 transmission, offering promise for further development and application in preventive interventions.^[^ ^101^ ^]^ In a subsequent investigation, Lara et al. conducted a study using the same polyvinylpyrrolidone‐coated silver nanoparticles (PVP‐coated AgNPs) in combination with neutralizing antibodies (NABs) targeting different epitopes of the HIV‐1 envelope glycoprotein. These antibodies included HIV‐1 gp41 monoclonal antibody 126–7, HIV‐1 gp120 PB1 Sub 2 antiserum, HIV‐1 gp120 PB1 gp120 antiserum, and HIV‐1 F425 B4e8 monoclonal antibody gp120, all of which act on trimers of the viral envelope glycoprotein. crucial for receptor binding and entry. The study evaluated the efficacy of NABs both in the presence and absence of AgNPs against both cell‐free (HIVIIIB) and cell‐associated (HVIIIB/H9) viruses. The results indicated that all NABs effectively suppressed cell‐free HIV‐1 infection in a dose‐dependent manner. However, when applied alone, NABs showed no inhibitory effects on infection by cell‐associated viruses. In contrast, silver nanoparticles (AgNPs) alone have demonstrated the ability to inhibit cell‐associated HIV‐1 infection. Furthermore, when combined with NABs, AgNPs showed an additive effect, leading to the effective inhibition of cell‐associated HIV‐1 infection. These results suggest a synergistic interaction between AgNPs and NAB in inhibiting cells associated with HIV infection in vitro, with dose‐dependent efficacy. This highlights the potential of combined therapeutic approaches involving AgNPs and NAB as a promising strategy to combat HIV infection.^[^ ^102^ ^]^ Fayaz et al. have successfully designed a polyurethane condom (PUC) coated with Ag‐NP, previously discussed in the context of its work against HSV. In addition to its efficacy against HSV, the effects of Ag‐NP‐coated PUC were evaluated against HIV‐1, using both (M)‐tropic and T lymphocyte (T)‐tropic strains of the virus. In experiments conducted with human HeLa cells, 293T cells, and C8166 T cells, no significant toxicity was observed after 3 h of contact with Ag‐NP‐coated PUC. Subsequently, exposure of HIV‐1 to Ag‐NP‐coated PUC resulted in a significant reduction in virus infectivity for both T‐tropic and M‐tropic strains. For the T‐tropic strain, most of the virus lost the ability to infect CD4^+^ T cells even after just 10 minutes of exposure. Light microscopy revealed the presence of only GFP^+^ cells without syncytium formation after exposure. Furthermore, for the M‐tropic virus, evaluation using the MAGI assay with HeLa‐β‐gal‐CD4^+^‐CCR5^+^ cells demonstrated a significant reduction in infectivity following exposure to Ag‐NP‐coated PUCs. After 30 minutes of exposure, the virus completely lost its infectivity. However, it was determined that PUC coated with AgNPs exhibited this inhibitory effect only during direct exposure. Viruses exposed to medium pre‐incubated with AgNPs‐coated PUC for 30 minutes still showed relatively high infectivity with C8166 CD4^+^ T cells, albeit at a 40% lower rate than uncoated condoms. This discrepancy was attributed to the low amounts of AgNPs released from the PUC over time.^[^ ^81^ ^]^ The study involved evaluating the replication of VSVG‐pseudotyped HIV‐1 SCR virions on HEK cells, with particular attention to the time course of viral replication. The concentration of P24 in the supernatant was quantified using the capture ELISA protocol (Biomerieux). The cytotoxicity of the new compound was evaluated using the XTT method. The results of the cytotoxicity test indicated that the test compound exhibited cytotoxicity at concentrations of 20, 40, 80, and 120 µm, with percentages of 17%, 23%, 36%, and 48%, respectively. However, all levels of Ag‐dendrimer nanoconjugates demonstrated effective HIV inhibition. Inhibition rates for concentrations of 120, 80, 40, and 20 µm were 78%, 67%, 52%, and 15%, respectively, with Nevirapine showing more severe toxicity. Despite the lowest inhibition rate observed at the concentration of 20 µm (15%), its cytotoxicity was only 17%. Therefore, this concentration of the new compound was deemed more suitable overall. Ag‐dendrimer conjugation was found to decrease P24 antigen expression on the surface of monocytes and lymphocytes, suggesting that virus inhibition occurs through the reduction of cell proliferation. This implies that Ag‐dendrimer conjugation does not directly target the virus but rather affects the host cells in which the virus replicates, leading to suppression of P24 production in lymphocytes.^[^ ^103^ ^]^ The potential of AgNPs in fighting HIV has been explored, using environmentally friendly synthesis methods. Kumar et al. achieved the biosynthesis of silver nanoparticles, ranging in size from 12 to 28 nm, through the reduction of Ag^+^ ions with an aqueous extract of leaves of the mangrove species *Rhizophora lamarckii*.^[^ ^104^ ^]^ Reverse transcriptase (RTase) plays a crucial role in the HIV replication cycle. Inhibition of this enzyme stops viral replication. Therefore, this study aimed to evaluate the RTase inhibitory activity of green‐synthesized silver nanoparticles. To evaluate this, RTase activity was quantified using a reverse transcriptase assay kit, employing purified recombinant HIV RTase. The inhibitory effect of AgNPs was evaluated in a concentration range between 0.25 and 1 mcg mL^−1^. Surprisingly, they showed significant inhibitory activity against HIV type 1 RTase, even at low concentrations. Specifically, the nanoparticles demonstrated an IC_50_ value of 0.4 mcg mL^−1^ against HIV‐1 RTase.^[^ ^104^ ^]^ Sharma et al. synthesized curcumin‐stabilized AgNPs (Cur‐AgNPs) with an average size of 45 nm. Their immunomodulatory properties and their potential as antiretroviral agents were evaluated.^[^ ^105^ ^]^ The potential toxicity of the nanomaterial was evaluated using THP‐1 cells via the CellTiter 96 non‐radioactive aqueous cell proliferation assay. The antiretroviral activity was tested against ACH‐2 cells latently infected with the human immunodeficiency virus (HIV‐1). ACH‐2 cells, at a concentration of 200000 mL^−1^, were treated with Cur‐AgNPs for 24–48 h. The impact of Cur‐AgNPs on HIV replication was assessed by measuring HIV‐1 LTR gene expression levels in ACH‐2 cells and quantifying HIV‐1 p24 levels in cell supernatants. Furthermore, the expression of proinflammatory cytokines, including IL‐1β, TNF‐α, and NF‐κB, was measured. The results indicated that treatment of ACH‐2 cells with Cur‐AgNPs showed no cytotoxic effects, significantly reducing the expression of HIV‐1 LTR by 73%, p24 by 57%, IL‐1β by 61%, TNF‐ α by 54%, IL‐6 by 68%, and NF‐κB by 79%, compared to untreated controls. Experimental controls, such as curcumin alone and conventional AgNPs coated with citric acid, did not elicit similar biological effects. The study concludes that Cur‐Ag NPs possess therapeutic potential as antiretroviral agents and direct immunomodulators by inhibiting the expression of proinflammatory mediators induced by HIV‐1 infections.^[^ ^105^ ^]^ Sampathkumar et al. used the microalgae species Nostoc sp., Lyngbya sp. and Phormidium sp. for the biosynthesis of AgNPs.^[^ ^106^ ^]^ The resulting nanoparticles were characterized as spherical and monodisperse, with sizes of 30 nm, 40 nm, and 80 nm for *Nostoc sp*., *Lyngbya sp*., and *Phormidium sp*., respectively. The antiviral activity of the synthesized AgNPs was evaluated against HIV‐1. The HIV test was conducted via a cell‐free method, in which AgNPs and control were used to pre‐treat the virus and subsequently incubated with MT‐2 cells or PBMCs. Nonoxynol‐9 served as a positive control. IC50 values for biogenic AgNPs were determined and HIV gag p24 content was quantified in the collected supernatants using ELISA. Molecular visualization of the HIV protein was achieved using Pymol. The analyses revealed that cell viability (IC50) in MT‐2 cells or PBMC for a concentration of 1 mg mL^−1^ of AgNPs biosynthesized from *Nostoc sp*., *Lyngbya sp*. and *Phormidium sp*. was found to be 74.9%, 62.3%, and 67.4%, respectively. It was suggested that the antiviral activity of the tested nanomaterials was attributed to their ability to cross lipophilic membranes and interact with proteins involved in apoptosis. Furthermore, it was observed that the antiviral potential of silver ions incorporated into the V3 loop of HIV‐GP120 is significant. CD4 cells, which bind to the chemokine receptor CXCR4 together with its ligand CXCL12, play a crucial role in defense against virus entry. It was observed that the nanoparticles were concentrated on the region of sulfur‐containing residues of GP‐120. Silver nanoparticles show strong affinity to the amino acids cysteine and methionine due to the presence of their free SH binding site on Cys‐296, Cys‐331, Met‐307, and Met 312.^[^ ^106^ ^]^ Although nanotechnology has shown encouraging outcomes in the treatment of HIV, research on this subject is still ongoing, and further clinical trials and investigations are required before it can be implemented as a successful therapeutic option (**Table** **4**).

**Table 4:** Characteristics and Activity of AgNPs Against HIV.

Type of nanoparticle	Synthesis method	Active concentrations	Refs.
Nanoparticles with distinct surface compositions: foamy carbon, poly(N‐vinyl‐2‐pyrrolidone) (PVP) and bovine serum albumin (BSA)	AgNPs were obtained from Nanotechnologies	< 25 µg mL^−1^	[21]
AgNPs coated with 0.2 wt% polyvinylpyrrolidone (PVP‐coated AgNPs)	AgNPs were acquired from NanoAmor	From 0.025 to 0.15 mg mL^−1^	[101]
Incorporation of AgNPs in polyurethane condom (PUC)	A 100‐mL solution of 7.0 mm aqueous trisodium citrate was prepared in a glass flask and heated to boiling while stirring. Subsequently, 1.0 mL of 0.1 M aqueous silver nitrate was added to initiate the reaction. The solution's color changed from colorless to yellow and eventually became turbid.	‐	[81]
AgNPs	The green biosynthesis of antiviral silver nanomaterials was accomplished by facilitating the reduction of Ag⁺ ions using the aqueous extract of mangrove leaves from *Rhizophora lamarckii*.	Between 0.25 and 1 mcg mL^−1^	[104]
AgNPs	AgNPs were synthesized from *Psidium guajava* by mixing varying concentrations of aqueous leaf extract with a 1 mm silver nitrate solution. The mixture was stirred in a boiling water bath for 15 to 20 minutes, and a color change indicating the formation of nanoparticles was observed after 24 h.	100 µl mL^−1^ (?)	[105]
AgNPs	A microalgal strain cultured in BG11 medium was harvested and processed to obtain a cell‐free extract, which was filtered to remove debris. The extract was divided into two flasks, with one containing 1 mm silver nitrate. Both flasks were incubated at 25 °C with shaking for 72 h, during which a brown color change indicated the successful synthesis of AgNPs	1 mg mL^−1^	[106]

### 2.5 Dengue Virus (DENV)

**Figure 5:**
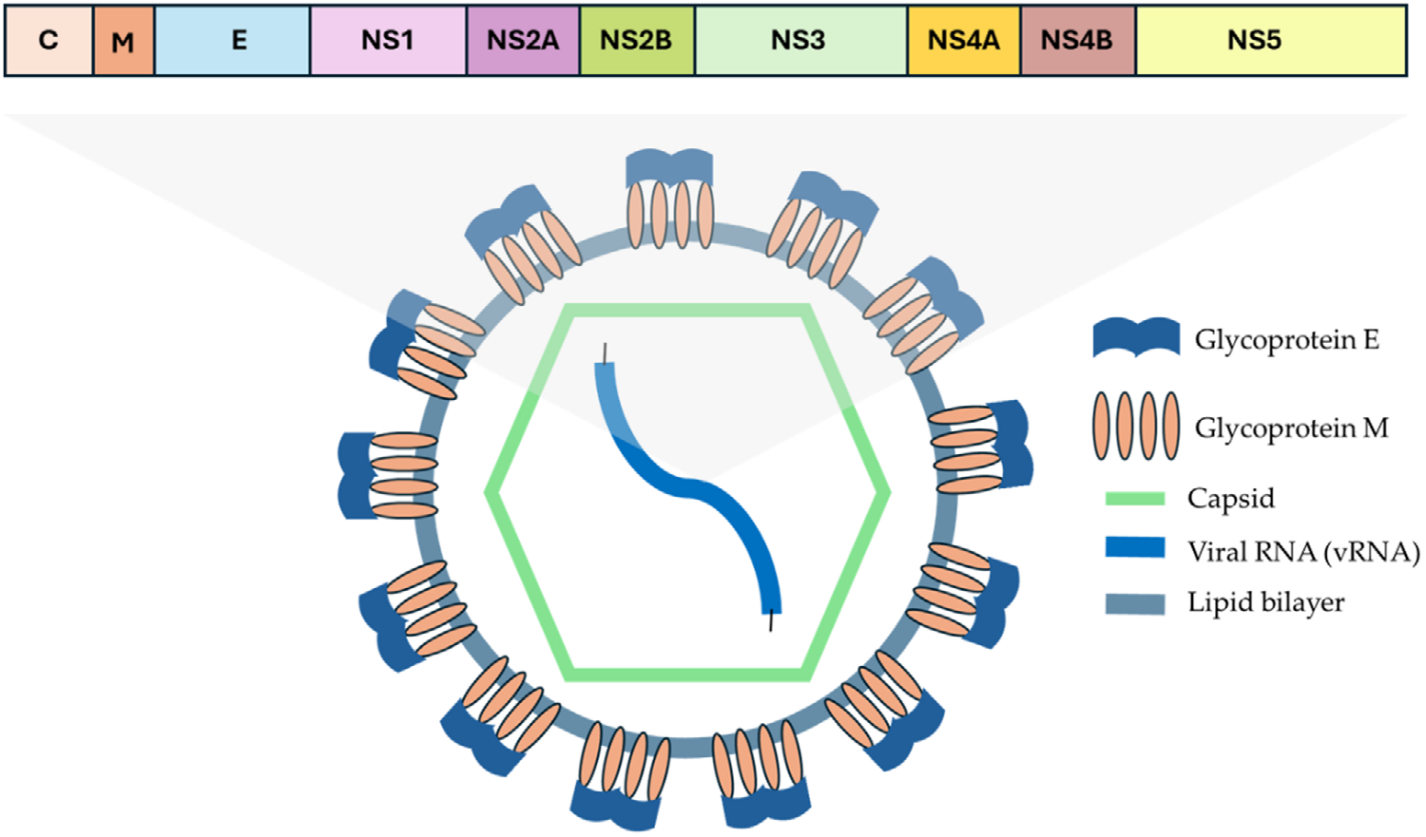
Dengue virion structure and genome organization.

Dengue virus (DENV), a positive‐sense, single‐stranded RNA virus of the Flaviviridae family, exists in four serotypes: DENV‐1, DENV‐2, DENV‐3, and DENV‐4. Structurally, it is a spherical virus about 50 nm in diameter. The central nucleocapsid core houses the viral RNA genome, which encodes ten genes. This core is surrounded by a host‐derived lipid envelope embedded with 180 copies of the envelope (E) and membrane (M) glycoproteins, which play key roles in host cell entry and viral assembly. The replication cycle begins with the E protein binding to host cell receptors, leading to receptor‐mediated endocytosis. Acidification in the endosome triggers a conformational change in the E protein, facilitating fusion of the viral envelope with the endosomal membrane and releasing the nucleocapsid into the cytoplasm. The viral RNA is then translated into a polyprotein, which is cleaved into structural and nonstructural proteins necessary for replication and assembly. New RNA genomes are synthesized by the NS5 protein, and immature virions are assembled in the endoplasmic reticulum. These virions mature in the Golgi apparatus and are released from the host cell via exocytosis, ready to infect new cells^[^ ^107^ ^]^ (**Figure** **5**). Although most dengue infections are subclinical or cause mild illness, serious complications, including deaths, can occur. The dengue virus affects a significant portion of the world's population. There is an urgent need for alternative interventions against vector‐borne diseases such as dengue infection, and environmentally produced metal nanoparticles show promise in dengue therapy.^[^ ^108^ ^]^ Green nano‐products which can be synthesized quickly, inexpensively, and don't require the use of hazardous substances, can used for such cases.^[^ ^109^ ^]^ Sujitha et al. biosynthesize AgNPs from *Moringa oleifera* seed extract, using it as a stabilizing and reducing agent. Their research proposes the application of green‐synthesized AgNPs as a new and powerful tool against the DEN‐2 serotype of dengue virus and its primary vector Aedes in Egypti.^[^ ^110^ ^]^ Biophysical characterization of the green AgNPs revealed a spherical morphology, with an average diameter of 100 nm. Evaluation of cytotoxicity using Vero cells demonstrated no significant changes in cell survival when exposed to various concentrations of AgNPs synthesized from green sources for up to 72 h. Furthermore, AgNPs showed in vitro antiviral activity against Dengue virus DEN‐2 in Vero cells. The viral titer, initially equal to 7 log10 TCID_50_ mL^−1^ in the control, decreased to 3.2 log10 TCID_50_ mL^−1^ after a single treatment with 20 µl mL^−1^ of AgNPs. After 6 h, the yield of DEN‐2 was 5.8 log10 PFU mL^−1^ in control, compared to 1.4 log10 PFU mL^−1^ post‐treatment with AgNPs (20 µl mL^−1^). Furthermore, AgNPs have demonstrated high efficacy against the dengue vector Aedes an Egypti.^[^ ^110^ ^]^ Murugan et al. conducted a study on the biosynthesis of AgNPs using *Bruguiera cylindrica* extract.^[^ ^111^ ^]^ These nanoparticles had a spherical shape with an average size between 30 and 70 nm. AgNPs exhibited toxicity with LC50 values ranging from 8.93 ppm for first‐stage larvae to 30.69 ppm for pupae of the Aedes aegypti vector. In vitro experiments further demonstrated that at a concentration of 30 µg mL^−1^, AgNPs significantly inhibited the production of the dengue viral envelope (E) protein in Vero cells and downregulated the expression of the dengue viral E gene.^[^ ^111^ ^]^ Later on, Murugan et al. biosynthesized AgNPs with an extract of alga *Centroceras clavulatum*. C. clavulatum‐synthesized AgNPs inhibited dengue (DEN‐2) viral replication in Vero cells. Notably, 50 µg mL^−1^ of green‐synthesized AgNPs showed no cytotoxicity on Vero cells while it reduced DEN‐2 viral growth by more than 80%; A dose of 12.5 µg mL^−1^ inhibited viral growth of more than 50%.^[^ ^112^ ^]^ For decades, *Carica papaya* leaf extract (CPLE) has been used to treat thrombocytopenia in dengue patients. Patil et al. evaluated the efficacy of AgNPs biosynthesized from *Carica papaya* leaf extract against the DEN‐2 virus. The average size of nanoparticles in the obtained colloidal system was 76.27 nm. Cytotoxic effects were evaluated on Vero CCL‐81 cells with the MTT assay. AgNPs based on *Carica papaya* leaf extract showed no cytotoxicity against Vero CCL‐81 cells. The nanoparticles were screened for antiviral activity under post‐infection conditions using the maximum non‐toxic dose (MNTD). The primary screening revealed that they exerted a 100% reduction in DENV‐2 titer compared to the control virus. Based on the results of the screening process, they were further studied under pre‐treatment, co‐treatment and post‐treatment conditions. Virus production after treatment was measured by real‐time RT‐PCR, immunofluorescence assays, and plaque‐forming unit assays. Consequently, a significant reduction in viral titer was observed: from 6.46 (VC) to 3.86 and 1.43 log10 mean FFU mL^−1^ at 50 µg mL^−1^ and 100 µg mL^−1^ respectively in the case of cells pretreated with AgNP. In the case of cotreatment, 100 µg mL^−1^ showed a reduction from 6.34 (VC) to 3.35 log10 FFU mL^−1^. When cells were treated with nanoparticles after infection, an approximately 99% reduction in viral foci was detected at 100 µg mL^−1^ from 6.48 (VC) to 0.76 log10 FFU mL^−1^ and a significant reduction at 50 µg mL^−1^ from 6.48 (VC) to 2.51 log10 FFU mL^−1^. Therefore, AgNPs biosynthesized from *Carica papaya* leaf extract have been shown to have good potential to combat the DEN‐2 virus.^[^ ^113^ ^]^ Dung et al. evaluate the effectiveness of chitosan‐stabilized AgNPs against the DEV‐1 virus. The results revealed that chitosan‐stabilized AgNPs could inhibit DENV‐1 by 96.67% and 98.34% at concentrations of 12.50 ppm and 25.00 ppm, respectively, without inducing toxic effects in Baby Hamster Kidney (BHKFcɣ) cells. In addition to their antiviral properties, the study confirmed the dual functionality of chitosan‐stabilized AgNPs, showcasing both antiviral and anti‐mosquito effects. The findings suggest that these nanoparticles could serve as an effective strategy for dengue prevention by targeting and inhibiting both mosquito larvae and adults. This dual functionality allows you to fight both the virus and the vector at the same time.^[^ ^114^ ^]^ Khan et al. used the aqueous extract of the rhizome and stem of *Alocasia odora* to biosynthesize AgNPs, synthesize stem aqueous extract‐AgNPs (SNP) and rhizome aqueous extract‐AgNPs (RNP), respectively.^[^ ^115^ ^]^ The researchers evaluated the in vitro cytotoxicity of these nanoparticles using the MTT assay on the U87‐MG human glioblastoma cancer cell line. Their results revealed that SNP had the highest cytotoxicity (43.40 µg mL^−1^), indicating its potent antitumor activity. Furthermore, these silver nanoparticles demonstrated significant cytopathic effects (CPE) against dengue virus type 2 (DENV‐2) in the Huh‐7 cell line. Overall, the study suggests that AgNPs synthesized from the aqueous extract of *Alocasia odora* possess promising virucidal potential and can be effectively used as antimicrobial agents^[^ ^115^ ^]^ (**Table** **5**).

**Table 5:** Characteristics and activity of AgNPs against DENV.

Type of nanoparticle	Synthesis method	Active concentrations	Refs.
AgNPs from *Moringa oleifera* seed extract	*Moringa oleifera* seeds were washed, dried and powdered. The extract was produced by boiling 5 g of powder in 100 mL of water, then filtered and stored at ‐4 °C. It was then mixed with 1 mm silver nitrate, resulting in the formation of silver nanoparticles.	20 µl mL^−1^	[110]
AgNPs from *Bruguiera cylindrica* extract	*B. cylindrica* leaves were washed, dried, and prepared as an extract by boiling 10 g of cut leaves in 100 mL of distilled water. The mixture was decanted, stored at −4 °C, and used within 5 days. When treated with 1 mm silver nitrate, the solution turned yellow‐brown, indicating the formation of AgNPs.	30 µg mL^−1^	[111]
AgNPs from extract of alga *Centroceras clavulatum*	The aqueous extract of *C. tomentosum* was obtained by boiling 10 g of chopped sponges in 100 mL of distilled water for 5 min, then filtered and stored at ‐4 °C. The extract was treated with 1 mm silver nitrate and incubated at room temperature. A yellow‐brown solution indicated the formation of AgNPs.	12.5 µg mL^−1^	[112]
AgNPs from *Carica papaya* leaf extract	Carica papaya leaves were heated in water for 25 minutes to prepare an extract. Various concentrations of this extract were mixed with 1 mm silver nitrate solution, leading to the formation of green AgNPs. After incubation at room temperature for 24 h, the solution changed from yellow to yellowish‐brown within 10–15 minutes, indicating the reduction of AgNO₃ to Ag^+^ ions.	50 µg mL^−1^	[113]
Chitosan‐stabilized AgNPs	Biological method	12.50 and 25.00 ppm	[114]
AgNPs	AgNPs were synthesized by adding 5 mL of plant aqueous extract to 45 mL of 1 mm silver nitrate solution in a flask and magnetically stirred on a hot plate at 80 °C for 4 h. Bioreduction of Ag ions into AgNPs was observed by the color change of the reaction mixture to dark reddish brown.	43.40 µg mL^−1^	[115]

### 2.6 Chikungunya Virus (CHIKV)

**Figure 6:**
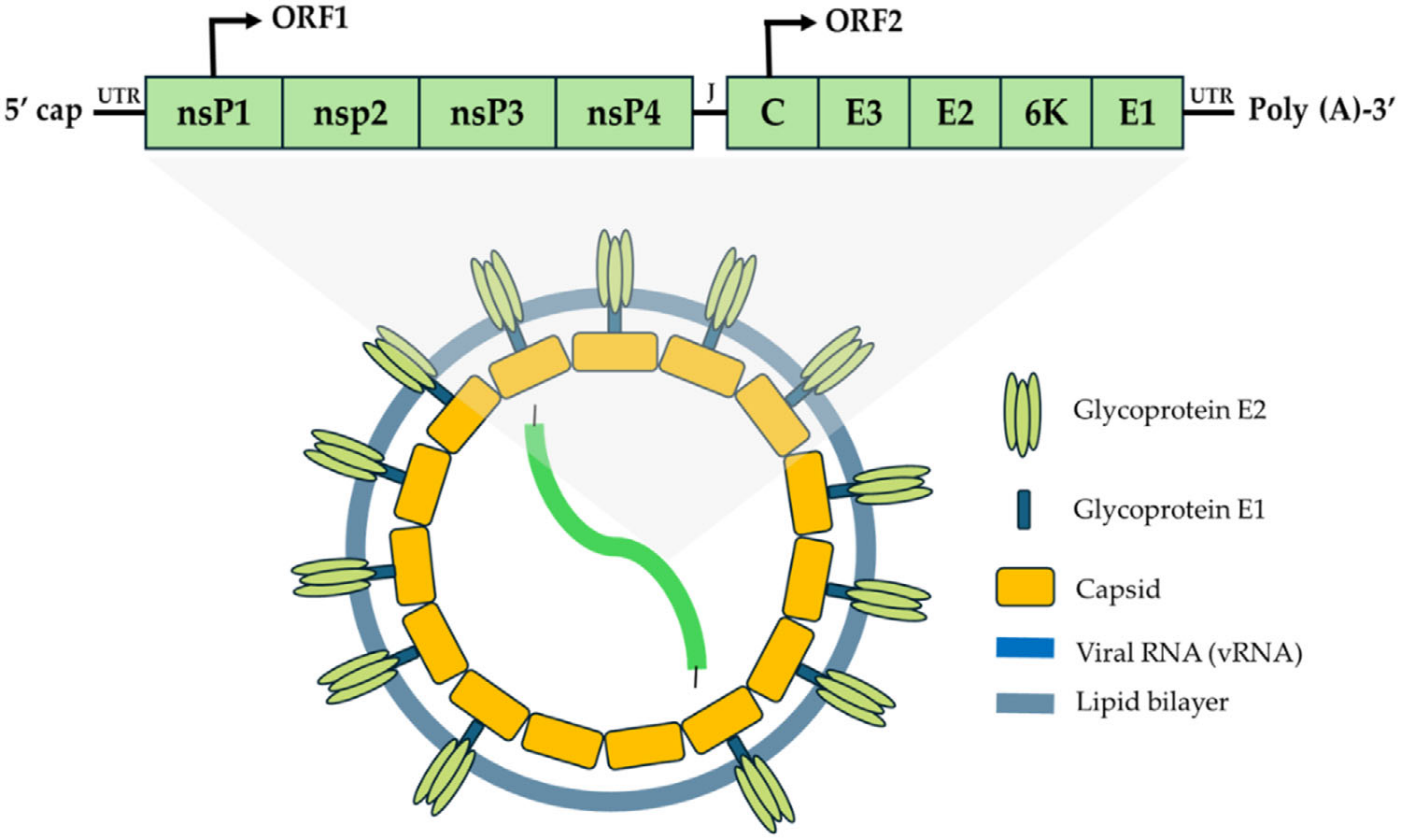
CHIKV structure and genome organization.

Chikungunya virus (CHIKV) is an enveloped, single‐stranded, positive‐sense RNA virus, which belongs to the genus *Alphavirus* in the *Togaviridae* family. The virus has a spherical structure, approximately 60–70 nm in diameter, composed of three main components: surface glycoproteins, a lipid envelope, and a nucleocapsid core. The surface glycoproteins E1 and E2 form trimeric spikes crucial for viral entry, with E1 facilitating membrane fusion and E2 binding to host cell receptors. The lipid envelope, derived from the host cell membrane, encases the nucleocapsid core, which is composed of the capsid (C) protein and the 11.8 kb RNA genome. The replication cycle begins with the virus binding to host cell receptors via E2, leading to internalization through endocytosis. Within the acidic endosome, E1 triggers fusion with the endosomal membrane, releasing the nucleocapsid into the cytoplasm. The viral RNA is then translated into non‐structural proteins that form a replication complex, synthesizing new RNA genomes. These genomes are either translated into structural proteins or packaged into new nucleocapsid cores. Finally, the nucleocapsid cores interact with E2 at the plasma membrane, triggering viral budding and release, allowing the infection of new cells (**Figure** **6**).^[^ ^116^ ^]^ Currently, no specific antiviral treatment or vaccine is available for chikungunya. However, various therapeutic options, including investigations into the use of silver nanoparticles, are being explored. Sharma et al. synthesized AgNPs from three different medicinal plant species, namely *Andrographis Paniculata*, *Phyllanthus niruri* and *Tinospora cordifolia*, and evaluated their antiviral activity against CHIKV. The size range of the obtained nanoparticles was as follows: *Andrographis Paniculata* AgNP: 70–95 nm; *Phyllanthus niruri* AgNP: 70–120 nm; and *Tinospora cordifolia* AgNP: 50–70 nm. The cytotoxicity of these nanoparticles was evaluated using Vero cells. AgNPs from *Andrographis Paniculata*, *Phyllanthus niruri* and *Tinospora cordifolia* showed a maximum non‐toxic dose (MNTD) value of 31.25, 125, and 250 µg mL^−1^, respectively. In an in vitro antiviral test based on the degree of inhibition of cytopathic effect (CPE), AgNPs from A. panicola showed the most significant efficacy. The antiviral activity of *Andrographis Paniculata* AgNPs was further validated by cell viability assay. CHIKV‐infected cells treated with *Andrographis Paniculata* AgNPs at the maximum non‐toxic dose and half of this showed a substantial increase in cell viability from 25.69% to 80.76% and 66.8%, respectively.^[^ ^117^ ^]^ Choudhary et al. have immobilized different nanoscale metals (Ag, Fe, and Zn) onto a raw *Citrus limetta* peel. For Ag, Fe, and ZnO, the average size of the nanoparticles was determined to be 5, 32, and 12 nm, respectively. Plaque assay, real‐time PCR, and indirect immunofluorescence assay (IFA) proved that all of these green‐synthesized nano‐biomaterials have successfully reduced the CHICKV virus titer and viral RNA level. ZnO had the lowest level of viral inhibition. Noteworthy, silver nanoparticles demonstrated the greatest antiviral effectiveness. At a minimal concentration of 0.087 µg the viral titer reduction was found to be 95%.^[^ ^118^ ^]^ The study conducted by Patil et al., which evaluated the antiviral efficacy of various C. papaya formulations against Dengue virus (DENV‐2), also extended its investigation to Chikungunya virus (CHIKV). Similar to the approach adopted with DENV‐2, the research evaluated the effectiveness of the formulations in different conditions such as pre‐treatment, co‐treatment, post‐treatment. The results of the screening phase indicated that, unlike its effect on DENV‐2, AgNPs did not demonstrate a significant impact on CHIKV. In contrast, powdered papaya leaves are effective against CHIKV.^[^ ^113^ ^]^ Sharma et al. evaluated the anti‐Chikungunya efficacy of AgNPs biosynthesized with *Psidium guajava* leaf extract. The average size of the obtained nanoparticles was in the range of 75–99 nm. Using Vero cell lines, the study examined maximum non‐toxic doses of both extracts and nanoparticles. Vero cells served as a platform to test the anti‐Chikungunya properties of the extracts and nanoparticles, with the MTT assay employed to evaluate their impact on cell viability. The maximum non‐toxic dose detected was 15 mg mL^−1^. The antiviral activity was evaluated based on the reduction of the cytopathic effects caused by the virus. Confluent monolayers were pretreated with the highest non‐toxic concentration of extracts and AgNPs. The plant extract showed a viability of 84.21%, while the silver nanoparticles produced a viability of 64.40%, compared to 24.03% for the positive control. The phytochemicals were molecularly docked against Chikungunya replication essential cysteine protease (nsP2) to elucidate the cause of their antiviral activity. Molecular docking analysis revealed that phytochemicals such as longifollen and quercetin exhibited the lowest binding energy with nsP2, indicative of a stable interaction^[^ ^119^ ^]^ (**Table** **6**).

**Table 6:** Characteristics and activity of AgNPs against CHIKV.

Type of nanoparticle	Synthesis method	Active concentrations	Refs.
AgNPs from the plant species *Andrographis Paniculata*, *Phyllanthus niruri*, and *Tinospora cordifolia*	The extracts of Andrographis Paniculata, *Phyllanthus niruri*, and *Tinospora cordifolia* were mixed with 1 mm silver nitrate solution and incubated in the dark for 24 h. Within 10 min of mixing, the solution color shifted from pale yellow to yellowish‐brown, indicating the reduction of silver nitrate to silver ions.	From 250 to 31.25 µg mL^−1^	[117]
Immobilized different nanoscale metals (Ag, Fe, and Zn) onto a raw *Citrus limetta* peel	Citrus limetta peels were cleaned, dried, and finely ground to a powder with a particle size of less than 1 mm. Silver nitrate was employed to facilitate the bioreduction of silver ions into AgNPs.	0.087 µg mL^−1^	[118]
AgNPs from *Psidium guajava* leaf extract	The synthesis of AgNPs using Psidium guajava leaf extract was carried out by adding varying concentrations of the aqueous leaf extract to 1 mm silver nitrate solution. The mixture was placed in a boiling water bath with continuous stirring for 15–20 min. After incubation for 24 h, the color change of the solution was observed.	15 mg mL^−1^	[119]

### 2.7 Other Enveloped Viruses

Beyond the previously discussed viruses, additional data highlight the effects of silver nanoparticles (AgNPs) on other enveloped viruses. A study by Lu et al. investigated the efficacy of these nanoparticles against hepatitis B virus (HBV), a double‐stranded DNA virus linked to chronic liver disease and hepatocellular cancer. AgNPs were synthesized from AgNO_3_ in HEPES buffer, producing nanoparticles with average diameters of ≈10 and 50 nm. The HepAD38 cell line was used as an infection model to evaluate the anti‐HBV activity of these particles in vitro, with cytotoxicity measured via the MTT assay. After 48 h of incubation with AgNPs, DNA was extracted from the supernatants and quantified using quantitative real‐time PCR. The study also assessed the uptake of AgNPs by HepAD38 cells. Results showed that AgNPs reduced extracellular HBV DNA production in HepAD38 cells by more than 50% compared to the control, with the 10 nm particles demonstrating higher efficacy. Also, gel mobility shift assays indicated that the 10 nm AgNPs bound to double‐stranded HBV DNA.^[^ ^120^ ^]^ Haggag et al. utilized extracts from *Lampranthus coccineus* and *Malephora lutea* for the green synthesis of silver nanoparticles (AgNPs) and assessed their antiviral properties. The antiviral activity of these AgNPs was tested against HSV‐1, as well as HAV‐10 and CoxB4 viruses. In addition to their effectiveness against HSV‐1, the AgNPs synthesized from *Lampranthus coccineus* exhibited significant antiviral activity against HAV‐10 and CoxB4. The maximum non‐toxic concentration (MNTC) of AgNPs derived from the hexane extracts of both plants averaged 46.87 µg mL^−1^. Notably, Lampranthus coccineus hexane nanoparticles demonstrated high antiviral efficacy, with IC_50_ values of 11.71 ng mL^−1^ for HAV‐10 and 12.74 µg mL^−1^ for CoxB4. In contrast, AgNPs synthesized from *Malephora lutea* extracts did not show antiviral activity. A docking study further predicted interaction patterns between the compounds in the extracts and specific viral enzymes, including herpes simplex thymidine kinase, hepatitis A 3c proteinase, and Coxsackievirus B4 3c protease.^[^ ^92^ ^]^ Shady et al. conducted a study to evaluate the efficacy of silver nanoparticles (AgNPs) against the hepatitis C virus (HCV), a major cause of liver disease worldwide. The AgNPs were synthesized using extracts from marine sponges, including Amphimedon spp., with particle sizes ranging from 8.22 to 14.30 nm. In silico modeling and subsequent in vitro tests revealed that these AgNPs effectively inhibited the HCV NS3 helicase and protease, with varying potency.^[^ ^121^ ^]^ Rogers et al. assessed the antiviral effectiveness of plasma gas‐synthesized AgNPs, ranging from 25 to 80 nm, and polysaccharide‐coated nanoparticles of the same sizes, against the monkeypox virus (MPV) using a plaque reduction assay. The study found that specific formulations, such as 25 nm polysaccharide‐coated and 55 nm uncoated AgNPs, significantly reduced plaque formation in a dose‐dependent manner without causing cytotoxicity.^[^ ^122^ ^]^ Speshock et al. explored the antiviral effects of both uncoated and polysaccharide‐coated AgNPs on the Tacaribe virus (TCRV), an arenavirus. Their findings demonstrated that AgNPs significantly reduced viral RNA production and progeny virus release, particularly when administered before or shortly after infection. The study also noted that the polysaccharide coating slightly diminished the nanoparticles' efficiency in inhibiting viral replication^[^ ^123^ ^]^ Gaikwad et al. studied silver nanoparticles (AgNPs) synthesized from cultures of various fungi, including *Alternaria* species, *F. oxysporum*, *Curvularia* spp., *C. indicum*, and *Phoma* spp. Their research focused on the antiviral effects of these AgNPs against parainfluenza virus type 3 (HPIV‐3), in addition to herpes simplex types 1 and 2, which had been discussed earlier. The study found that AgNPs produced by *F. oxysporum* and *Curvularia* spp., with particle sizes of 4–13 nm and 5–23 nm respectively, exhibited 80–90% inhibition against HPIV‐3 and HSV‐1, with minimal cytotoxicity in Vero cells. In contrast, AgNPs synthesized from *Alternaria* and *Phoma* species showed lower antiviral activity, highlighting the size‐dependent nature of AgNPs in inhibiting HPIV‐3 through virus‐cell contact interference.^[^ ^89^ ^]^ Trefry and Wooley explored the antiviral effects of AgNPs around 25 nm in size against the Vaccinia virus. Their experiments revealed that while AgNPs did not directly kill the virus, they effectively inhibited its replication by preventing viral entry into cells. The half‐maximal inhibitory concentration for blocking viral entry was found to be 27.4±3.3 mg mL^−1^. They also identified that macropinocytosis played a crucial role in the antiviral mechanism, as blocking this process significantly reduced the inhibitory effect.^[^ ^124^ ^]^ *3/‐** (PPRV). The nanoparticles, averaging 20 nm in size, were found to significantly reduce PPRV replication at a concentration of 11.11 mg mL^−1^ by interfering with the virus entry phase. Transmission electron microscopy showed that AgNPs interacted with both the virion surface and core, blocking the virus from entering target cells, though without directly killing the virus.^[^ ^125^ ^]^ Yang et al. investigated the antiviral effects of curcumin‐biosynthesized silver nanoparticles (cAgNPs) against respiratory syncytial virus (RSV), a significant cause of respiratory infections. The cAgNPs, averaging 11.95 nm in size, were evaluated for biocompatibility in Hep‐2 cells. Through various tests, including viral titer and plaque assays, the study found that cAgNPs effectively inactivated RSV by blocking its entry and interfering with replication, outperforming conventional AgNPs and curcumin alone. Pretreatment with cAgNPs was particularly effective in preventing RSV attachment to cells and reducing viral infectivity.^[^ ^126^ ^]^ Morris et al. explored the antiviral and immunoregulatory properties of 10 nm PVP‐coated AgNPs against RSV. In vitro experiments on HEp‐2 and A549 cells showed a dose‐dependent reduction in RSV replication, with the most potent effect observed at 50 µg mL^−1^. In vivo studies using BALB/c mice revealed that AgNP treatment significantly reduced viral titers and pro‐inflammatory markers, indicating strong antiviral efficacy^.[^ ^127^ ^]^ Borrego et al. assessed the antiviral activity of AgNPs functionalized with polyvinylpyrrolidone (Argovit) against the Rift Valley fever virus (RVFV). While in vitro studies showed limited efficacy due to cytotoxicity, in vivo experiments demonstrated that higher doses of AgNPs significantly reduced viral infectivity, delayed disease onset, and improved survival rates in mice^[^ ^128^ ^]^ Chen et al. focused on graphene oxide sheets functionalized with AgNPs (GO‐Ag) against feline coronavirus (FCoV). The GO‐Ag nanoparticles, sized between 5 and 25 nm, exhibited lower toxicity and higher antiviral activity than GO alone. GO‐Ag achieved a 25% reduction in FCoV infection, compared to the 16% inhibition by GO alone, demonstrating enhanced antiviral properties^[^ ^129^ ^]^ Wan et al. investigated the impact of silver nanoparticles (AgNPs) on tumor cells linked with Kaposi's sarcoma‐associated herpesvirus (KSHV) and Epstein‐Barr virus (EBV). They found that AgNPs, averaging 25 nm in size, increased cytotoxicity in cells latently infected with these viruses by triggering viral lytic replication. This was due to enhanced reactive oxygen species (ROS) production and autophagy. AgNPs also effectively inhibited primary KSHV infection and reduced plaque formation and tumor growth in a mouse model of KSHV‐associated primary effusion lymphoma.^[^ ^130^ ^]^ Saadh examined AgNPs synthesized from green tea leaf extract for their antiviral activity against Newcastle disease virus (NDV). AgNPs showed inhibition of NDV at concentrations from 40 to 1280 mg mL^−1^, though they became cytotoxic to Vero cells at 640 mg mL^−1^. Noncytotoxic doses of AgNPs significantly blocked NDV entry and replication, with notable reductions in viral titers in eggs, suggesting AgNPs could be a promising therapeutic for NDV.^[^ ^131^ ^]^ Dung et al. explored the use of AgNPs against African swine fever virus (ASFV). Their study showed that AgNPs, applied at a 25 ppm solution, reduced microbial contamination in pigs and completely inhibited ASFV at a titer of 103 HAD50. AgNPs were non‐toxic to porcine alveolar macrophage cells at 0.78 ppm, highlighting their potential as effective disinfectants and antiviral agents for ASFV control.^[^ ^132^ ^]^ Garcia‐Serradilla and Risco assessed AgNPs against the Bunyamwera virus (BUNV) and compared them to ribavirin. AgNPs significantly reduced BUNV infection, decreasing extracellular virus production by up to three orders of magnitude. They localized in mitochondria, nuclei, and lysosome‐like organelles, demonstrating their potential as effective antiviral agents against BUNV infection.^[^ ^133^ ^]^ Hamouda et al. tested AgNPs‐treated polyester/viscose wipes against MERS‐CoV. They synthesized AgNPs using various methods and found the photochemically reduced AgNPs with polyacrylic acid to be the least cytotoxic while showing 48.3% viral inhibition at a concentration of 0.0625 µL. AgNPs prepared with cotton fabric exhibited even greater antiviral activity with 51.7% inhibition, suggesting their potential as effective tools for combating MERS‐CoV.^[^ ^134^ ^]^

Elshazly et al. evaluated AgNPs derived from Ginkgo biloba leaf extract against MERS‐CoV and human coronavirus 229E (HCoV‐229E). They found that AgNPs reduced MERS‐CoV replication by 61.09% and HCoV‐229E replication by 81.05%, indicating their potential effectiveness in treating coronavirus infections.^[^ ^135^ ^]^ Saadh further studied AgNPs against Goatpox virus (GTPV) and avian infectious bronchitis virus (IBV). AgNPs, biosynthesized from Piper beetle leaf extract, showed significant antiviral effects against GTPV, reducing viral genome copy number. They also inhibited IBV by preventing virus entry into cells and reducing viral RNA levels, highlighting their potential against poultry infections (**Table** **7**).^[^ ^131^, ^136^ ^]^

**Table 7:** Characteristics and activity of AgNPs against other enveloped virus.

Type of nanoparticle	Synthesis method	Viruses	Active concentrations	Refs.
AgNPs from *Lampranthus coccineus* and *Malephora lutea* extracts	AgNPs were synthesized by macerating 10 g of air‐dried powder from the aerial parts of *L. coccineus* and *M. lutea* in 100 mL of distilled water at 60 °C for 30 min. After filtration, the extract was mixed with a 1 mm silver nitrate solution in a 2:10 ratio and heated in a water bath at 60 °C for 10 minutes to facilitate the AgNPs biosynthesis.	HSV‐1 HAV‐10 and CoxB4 viruses	46.87 µg mL^−1^ 11.71 ng mL^−1^ 12.74 µg mL^−1^	[92]
AgNPs from marine sponges, such as *Amphimedon spp*	A 0.002 g extract of *Amphimedon* spp. and its petroleum ether fraction were dissolved in DMSO and 0.4 mL of each solution was mixed with 10 mL of 1 mm AgNO₃ at room temperature for the formation of AgNPs.	HCV	Against the HCV NS3 helicase with IC_50_ values of 0.11±0.62 and 1.52±1.18 and against HCV proteases with IC_50_ 2.38±0.57 and 9.76±0.58	[121]
AgNPs	AgNPs were synthesized with gas plasma	MPV	From 100 to 12.5 mg mL^−1^	[122]
Uncoated and coated AgNPs with polysaccharide	AgNPs were synthesized by reducing silver ions in solution using acacia gum as a polysaccharide‐reducing agent.	TCRV	100, 75, and 50 µg mL^−1^	[123]
AgNPs from conditioned media of cultures of *Alternaria species*, *F. oxysporum*, *Curvularia* spp., *C. indicum* and *Phoma* spp.	Fungal biomass of Dryopteris spp., *Musa paradisiaca*, *Catharanthus roseus*, *Selaginella bryopteris*, and *Syzygium cumini* species was grown in potato dextrose broth at 28 °C for 72 h. The biomass was filtered, washed, and suspended in distilled water for 48 h. The resulting cell filtrate was then treated with 1 mm AgNO₃ for the formation of AgNPs.	HPIV‐3	From 100 to 1 µg mL^−1^	[89]
AgNPs	–	Vaccinia virus	27.4/−3.3 mg mL^−1^	[124]
AgNPs biosynthesized using curcumin (cAgNPs)	AgNPs were synthesized by reducing silver ions using curcumin as the reducing agent.	RSV	From 0.24 to 0.008 nm	[126]
PVP‐coated AgNPs	AgNPs were acquired by NanoComposix Inc.	RSV	50 µg mL^−1^	[127]
AgNPs functionalized with polyvinylpyrrolidone (Argovit)	AgNPs Argovit was provided by Vector‐Vita Ltd	RVFV	4.8 µg mL^−1^	[128]
Graphene oxide (GO) sheets functionalized with AgNPs (GO‐Ag)	Graphene oxide powders were suspended in a solution of 35 mL of 0.5 m AgNO₃ and 70 mL of ethylene glycol. This mixture was subjected to pulsed microwave‐assisted synthesis by heating in a microwave oven at 160 °C for 5 min to promote the growth of silver seeds on the surface of graphene oxide.	FCoV	1,56 mg mL^−1^	[129]
AgNPs	AgNPs are produced by liquid chemical synthesis, using polyvinylpyrrolidone as a stabilizing agent.	KSHV and EBV	5 and 10 µg mL^−1^	[130]
AgNPs from green tea leaf extract	An aqueous extract of green tea leaves (5 g/100 mL) was prepared and 10 mL of it was combined with 5 mm AgNO3 solution. The mixture was left at room temperature (25 °C) for 4 h and centrifuged at 10 000 rpm for 10 min. The resulting pellet was washed twice with distilled water (7000 rpm for 2 min) and dried in a vacuum desiccator.	NDV	From 40 to 1280 mg mL^−1^	[131]
AgNPs‐treated polyester/viscose wipes	AgNPs were prepared using four different methods: 1) trisodium citrate with cotton yarn as a reducing agent, 2) silver nanoparticles (AgNPs) synthesized using an aqueous PVA solution with glucose, 3) trisodium citrate with cotton fabric as a reducing agent, and 4) a photochemical reaction involving polyacrylic acid and silver nitrate solution.	MERS‐CoV	From 3.868 to 0.0625 µL	[134]
AgNPs from *Ginkgo biloba* leaf extract	Two grams of Ginkgo leaf powder were dissolved in 100 mL of deionized water and sonicated at 80 °C for 40 min. The aqueous extract was then treated with 5% AgNO_3_ solution dropwise (ratio 1:2) at 80 °C and 3000 rpm for one hour, producing AgNPs. The final solution was dark brown, indicating the successful formation of AgNPs	MERS‐CoV HCoV‐229E	Inibition of replication at 125 mg mL^−1^; Reduction of replication at 15.62 mcg mL^−1^	[135]
AgNPs	The *P. betle* aqueous extract was prepared by mixing 10 g of dried leaves with 100 mL of purified water and incubating at 8 °C for 72 h. AgNPs were produced by combining 10 mL of the extract with 5 mmol silver nitrate, incubating at 25 °C for 4 h, followed by centrifugation. The pellet was washed, vacuum‐dried, and stored at room temperature for analysis.	GTPV IBV	−100 µg mL^−1^ −10 µg mL^−1^	[136, 137]

## 3 Toxicity of Secondary Silver Particles

Silver nanoparticles (AgNPs) undergo a variety of chemical and physical transformations in biological and environmental systems, resulting in the formation of secondary silver by‐products such as silver ions (Ag⁺), nanoparticle aggregates and agglomerates, silver sulfide (Ag₂S), silver chloride (AgCl), oxidized silver species (Ag₂O), and surface‐coated silver nanoparticles.^[^ ^138^ ^]^ These transformations have a significant impact on the stability, reactivity, antimicrobial efficacy, and toxicity of AgNPs, making their health and safety effects a critical area of study. One of the most documented transformations is the dissolution of AgNPs into silver ions (Ag⁺) in aqueous environments, particularly in biological fluids and tissues.^[^ ^139^ ^]^ Ag⁺ release is influenced by factors such as pH, temperature, ionic strength, and interactions between biomolecules, which in turn influence the bioavailability and toxicity of silver. AgNP aggregation and agglomeration also occur over time due to electrostatic and van der Waals interactions, particularly in biological media, altering their size, surface reactivity, and cellular interactions.^[^ ^140^ ^]^ In sulfur‐rich environments, such as biological tissues and wastewater, AgNPs react with sulfur‐containing compounds such as glutathione, cysteine, and hydrogen sulfide, forming silver sulfide (Ag₂S) nanoparticles. These Ag₂S particles are more stable and less bioavailable than their ionic counterparts, potentially reducing toxicity.^[^ ^141^ ^]^ In chloride‐rich environments, such as blood and gastric fluids, AgNPs react with chloride ions (Cl⁻), leading to the formation of silver chloride (AgCl) nanoparticles, which are relatively insoluble and exhibit lower toxicity^.[^ ^142^ ^]^ Similarly, in air and aqueous media, AgNPs undergo oxidation, forming silver oxide (Ag₂O) species, which can alter their antimicrobial properties and biological effects.^[^ ^143^ ^]^ In addition, AgNPs frequently undergo surface modifications due to interactions with biomolecules, proteins, and environmental macromolecules, leading to the formation of a protein corona. This protein coating modulates their cellular uptake, immune response, and overall biological behavior, influencing their toxicity and biocompatibility.^[^ ^144^ ^]^ These transformations play a crucial role in shaping the toxicity, stability, and biomedical applications of AgNPs, reinforcing the need for further research into their safety and long‐term effects in both the medical and environmental fields.^[^ ^145^ ^]^

### 3.1 Toxicity at the Microbial Level

AgNPs exhibit potent broad‐spectrum antimicrobial properties, effectively targeting bacteria, fungi, and viruses. Their antibacterial mechanism involves disruption of the cell membrane, interaction with thiol groups in proteins, and inhibition of key metabolic enzymes, ultimately leading to cell dysfunction and bacterial death. However, the non‐selective antimicrobial action of AgNPs extends beyond pathogenic bacteria, significantly altering the gut microbiota (GM), potentially leading to dysbiosis and adverse health consequences. The widespread use of AgNPs in various consumer products and medical applications increases the risk of human ingestion and systemic exposure.^[^ ^146^ ^]^ Several studies have shown that secondary silver particles, such as silver ions (Ag⁺), silver sulfide (Ag₂S), silver chloride (AgCl), and oxidized silver species (Ag₂O), contribute to microbial toxicity.^[^ ^147^ ^]^ Ag⁺ ions, in particular, play a crucial role in antimicrobial activity but also induce oxidative stress, compromising beneficial microbial populations in the gut.^[^ ^148^ ^]^ Wang et al. reported that oral administration of AgNPs in mice significantly altered the composition of the gut microbiota, leading to a reduction in Firmicutes and Bacteroidetes, two key bacterial phyla essential for gut homeostasis.^[^ ^149^ ^]^ Similarly, exposure of zebrafish to AgNPs functionalized with intestinal abalone hydrolysates resulted in a decrease in Flavobacterium, Halomonas, *Pseudomonas alcaligenes*, and *Plesiomonas shigelloides*, while promoting the overgrowth of Actinobacteria, Burkholderia, Proteobacteria, and Sphingomona.^[^ ^150^ ^]^ The impact of AgNPs on the gut microbiota extends beyond microbial composition, influencing metabolic functions and immune homeostasis. A study by Wang et al. examined the effects of AgNPs after oral administration (0.5 mg kg^−1^ and 2.5 mg kg^−1^ for 14 and 28 days), revealing that short‐term exposure inhibited the proliferation of Gram‐negative bacteria and reduced the diversity of the gut microbiota. Interestingly, microbial communities showed partial recovery after 28 days, suggesting some degree of resilience; however, significant metabolic alterations persisted, including increased levels of 1H‐indole‐3‐carboxylic acid and elevated serotonin in the gut and bloodstream, highlighting the potential for long‐term host metabolic disruption.^[^ ^151^ ^]^ Disruptions to the gut microbiota may impair immune regulation, as commensal bacteria play a crucial role in modulating toll‐like receptors (TLRs), activating T cells, and regulating antimicrobial peptide production via the NF‐κB signaling pathway. AgNP‐induced microbial alterations may predispose the host to immune dysregulation, increased susceptibility to infections, and chronic inflammatory states.^[^ ^152^ ^]^ Due to the widespread use of AgNPs, further research is needed to assess their long‐term impact on microbiota stability and immune function. Although AgNPs remain a valuable antimicrobial tool, their potential to drive bacterial resistance and disrupt gut microbial ecosystems requires careful regulation of their use. Strategies such as targeted functionalization of AgNPs, controlled dosing, and biocompatible coatings may help mitigate adverse effects while maintaining antimicrobial efficacy.^[^ ^153^ ^]^ Future studies should focus on assessing long‐term microbiome alterations to balance the clinical benefits of AgNPs with the need to preserve microbial homeostasis and host immune integrity.

### 3.2 Toxicity on Mammalian Cells

Although AgNPs exhibit significant antimicrobial potential, their effects on mammalian cells raise serious safety concerns. Secondary silver particles can increase bioavailability and toxicity, leading to oxidative stress, mitochondrial dysfunction, DNA damage, and inflammatory responses that ultimately result in apoptosis or necrosis.^[^ ^153^ ^]^

A major mechanism of AgNP toxicity is the overproduction of reactive oxygen species (ROS), which disrupts cellular homeostasis and activates the inhibitory kappa B kinase (IKK)/nuclear factor‐kappa B (NF‐κB) signaling pathway. This leads to cytoskeletal disruption, impaired DNA repair, and activation of p53‐ and mitochondria‐dependent apoptosis pathways, ultimately inducing cell death. Even at low doses, AgNPs can alter energy metabolism, cell cycle, and gene expression, particularly in fibroblasts, contributing to long‐term cytotoxic effects.^[^ ^20^ ^]^

AgNPs also disrupt cell membrane integrity and ion transport by interacting with membrane proteins and ion channels, especially Na⁺ and K⁺ channels, causing membrane potential imbalances. Their interaction with sulfhydryl (─SH) groups in membrane proteins impairs barrier function and material exchange, leading to membrane disruption and necrosis. Structural changes in membrane phospholipids, peptides, and sugar‐phosphate groups, detected using attenuated total reflection Fourier transform infrared (ATR‐FTIR) spectroscopy, indicate that AgNPs severely affect membrane stability and permeability.^[^ ^154^ ^]^

Once internalized, AgNPs undergo endocytosis through macropinocytosis, clathrin‐mediated, caveolae‐mediated, and non‐clathrin/non‐caveolin pathways. After cellular uptake, AgNPs migrate to mitochondria and nucleus, where they cause DNA fragmentation, genotoxicity, mitochondrial dysfunction, and apoptosis. Furthermore, AgNPs accumulate in lysosomes, where acidic conditions promote oxidative dissolution, releasing silver ions (Ag⁺) that increase ROS levels, damage lysosomal membranes, and trigger cytoplasmic leakage of toxic nanoparticles, leading to increased cytotoxicity.^[^ ^155^ ^]^

Mitochondrial dysfunction is another critical factor in AgNP toxicity. Exposure to AgNPs induces mitochondrial swelling, increased ROS production, and loss of mitochondrial membrane potential, leading to cytochrome C release and activation of apoptotic pathways. Studies show that AgNPs positively regulate the mitochondrial fission protein p‐Drp1 while negatively regulating the mitochondrial biogenesis regulator PGC‐1α, disrupting mitochondrial function and biogenesis. Furthermore, AgNPs alter mitochondrial redox homeostasis, leading to protein oxidation, cellular stress, and eventual apoptosis.^[^ ^156^ ^]^

Furthermore, AgNP exposure induces endoplasmic reticulum (ER) stress, disrupting protein folding and triggering the unfolded protein response (UPR) via the PERK, IRE1, and ATF6 signaling pathways. In drug‐resistant cells, AgNPs interfere with P‐glycoprotein (P‐gp) expression, impairing protein folding and contributing to multidrug resistance. AgNPs also reduce ER calcium levels, which affects calnexin/calreticulin cycles and promotes ER stress‐mediated apoptosis.^[^ ^157^ ^]^

Autophagy, a cellular defense mechanism, is initially activated by AgNP exposure. However, prolonged exposure results in defective autophagosome‐lysosome fusion, increasing cellular toxicity. Furthermore, AgNPs interfere with the ubiquitin‐proteasome pathway, disrupting protein degradation and cellular homeostasis, further amplifying cytotoxic effects.^[^ ^154^ ^]^

Lysosomal dysfunction is a major consequence of AgNP exposure, as the nanoparticles accumulate in lysosomes where acidic environments promote oxidative dissolution, leading to ROS generation and lysosomal disruption. This results in the release of proteolytic enzymes, causing autophagic stress, inflammation, and cell death. Furthermore, AgNPs activate the NOD‐like receptor protein 3 (NLRP3) inflammasome, leading to caspase‐1 activation and secretion of pro‐inflammatory cytokines (IL‐1β, IL‐18), contributing to chronic inflammation and tissue damage.^[^ ^158^ ^]^

The cytotoxic effects of AgNPs are influenced by factors such as size, surface charge, coating, and duration of exposure. Understanding these mechanisms is crucial to minimize the toxicity of AgNPs and optimize their biocompatibility for biomedical applications. Future research should focus on safer AgNP formulations, targeted delivery systems, and long‐term toxicity assessments to ensure their safe integration into medical and industrial settings.

## 4 Discussion

Enveloped viruses pose a significant and ongoing threat to public health, as demonstrated by their role in major pandemics and epidemics. Filoviruses such as Ebola and Marburg viruses have caused major hemorrhagic fever outbreaks, while coronaviruses have been responsible for global health crises, including the SARS outbreak in 2003, the MERS outbreak in 2012, and the COVID‐19 pandemic.^[^ ^159^ ^]^ Considering the high transmission, rapid mutation rates, and the lack of broad‐spectrum therapies, the potential for future pandemics remains high.^[^ ^160^ ^]^ This underscores the urgent need for new antiviral drugs and the implementation of effective preventive strategies. Widespread impact of enveloped viruses broad‐spectrum antiviral therapies are lacking, requiring innovative approaches to combat dangerous pathogens.

AgNPs are promising candidates for combating enveloped viruses due to their multifaceted mechanisms of action. First, AgNPs disrupt the viral envelope by interacting with its lipid bilayer, resulting in structural damage and loss of viral.^[^ ^54^ ^]^

In addition, AgNPs can bind to viral surface glycoproteins, preventing viral attachment and entry into host cells.^[^ ^21^ ^]^ In addition, AgNPd can interfere with the cellular pathways of the virus and alter the cellular response.^[^ ^161^ ^]^ They interact with the viral genome and inhibit its replication. They can also influence protein production or other cellular factors important for viral replication.^[^ ^137^ ^]^ These mechanisms make AgNPs a reliable and versatile option in the fight against enveloped viruses.

Several advantages favor their use including broad‐spectrum antiviral activity against influenza viruses (H1N1, H7N3, H9N2), coronaviruses (SARS‐CoV‐2), herpes viruses, human immunodeficiency virus (HIV), hepatoviruses and dengue viruses. Due to their multiple mechanisms of action, AgNPs show a lower tendency to induce viral resistance, compared to conventional antiviral drugs. Furthermore, they exhibit synergistic effects when used in combination with existing antiviral treatments, potentially improving efficacy while reducing the required dosage of standard antiviral agents^[^ ^162^ ^]^ Despite their potential, large‐scale clinical implementation of AgNPs remains limited due to toxicity concerns. Factors such as the size, shape, coating, and method of production of AgNPs significantly influence their biocompatibility and safety profileAddressing these challenges is crucial for the safe and effective clinical use of AgNPs and ongoing clinical trials are evaluating their efficacy and safety in several antiviral applications.^[^ ^163^ ^]^ Clinical research has explored the application of AgNPs in the treatment of various viral infections, including human papillomavirus (HPV), SARS‐CoV‐2, and herpes simplex virus (HSV). A Phase I/II clinical trial (NCT02338336) conducted by Nowarta Biopharma in 2015 evaluated the efficacy of Nowarta110, a fig extract formulation with AgNPs for the treatment of plantar warts (HPV‐related). Among 54 patients enrolled, 28 received Nowarta110, of which 18 achieved complete clearance of warts while 10 showed a reduction in lesions of 20–80%. The study found Nowarta110 to be highly effective, well tolerated, and with minimal side effects, justifying its Phase III study in 2024.^[^ ^164^ ^]^

A prospective randomized study (NCT04894409) during the COVID‐19 pandemic evaluated Argovit, an AgNP‐based antiviral formulation, for the prevention of infections in high‐risk healthcare workers. Among 231 participants, those who used Argovit mouthwashes and nasal rinses (1% w/w AgNP) for 9 weeks had a significantly lower infection rate (1.8%) compared to the control group (28.2%), demonstrating an 84.8% risk reduction. These results suggest that AgNP‐based nasal and oral rinses may help reduce SARS‐CoV‐2 transmission in high‐risk populations.^[^ ^165^ ^]^

A clinical trial at Hôpital Universitaire Sahloul (Tunisia) (NCT04978025) evaluated AgNP therapy in patients infected with SARS‐CoV‐2, administering AgNPs via inhalation (5 mL nebulized dose) or oral solution (30 mL dose) three times daily for 5 days. Control groups received a placebo orally or via inhalation, and patient outcomes were assessed on day 10, with adverse effects monitored for one month. The study showed that AgNPs could reduce disease severity and improve recovery rates in COVID‐19 patients.^[^ ^166^ ^]^

Although these clinical studies highlight the significant potential of AgNPs in antiviral applications, several challenges remain before their widespread adoption. AgNP toxicity is a major concern, as high doses or prolonged exposure can induce oxidative stress, inflammation, and cytotoxicity. Strategies to improve biocompatibility, such as surface modifications, controlled‐release formulations, and targeted delivery systems, are critical to mitigate these risks. Another challenge is the standardization of AgNP formulations, as differences in synthesis methods, coatings, and particle sizes can lead to variability in efficacy and safety profiles.^[^ ^167^ ^]^

In conclusion, AgNPs have shown great promise as broad‐spectrum antiviral agents, demonstrating efficacy against multi‐enveloped viruses while minimizing the risk of viral resistance. Their unique mechanisms of action make them a compelling alternative to existing antiviral therapies. However, further research and development is needed to fully realize their potential, particularly in optimizing biocompatibility, safety, and clinical applicability. By refining formulations to improve efficacy and minimize toxicity, AgNPs could become a valuable addition to the healthcare system, offering significant therapeutic benefits for the prevention and treatment of viral infections.^[^ ^160^ ^]^

## Conflict of Interest

The authors declare no conflict of interest.

## References

[1] N. H. L. Leung , Nat. Rev. Microbiol. 2021, 19, 528. 33753932 10.1038/s41579-021-00535-6 PMC7982882

[2] E. S. Bailey , J. Y. Choi , J. K. Fieldhouse , L. K. Borkenhagen , J. Zemke , D. Zhang , G. C. Gray , Evol., Med., Public Health 2018, 2018, 192. 30210800 10.1093/emph/eoy013 PMC6128238

[3] I. Chakrabartty , M. Khan , S. Mahanta , H. Chopra , M. Dhawan , O. P. Choudhary , S. Bibi , Y. K. Mohanta , T. B. Emran , Ann. Med. Surg. 2022, 79, 103985. 10.1016/j.amsu.2022.103985 PMC9188442 35721786

[4] K. Roe , Scand. J. Immunol. 2020, 92, 12938. 10.1111/sji.12928 PMC7361161 32640050

[5] M. Ghattas , G. Dwivedi , M. Lavertu , M. G. Alameh , Vaccines 2021, 9, 1490. 34960236 10.3390/vaccines9121490 PMC8708925

[6] F. Zoulim , F. Lebossé , M. Levrero , Curr. Opin. Virol. 2016, 18, 109. 27318098 10.1016/j.coviro.2016.06.004

[7] K. Wisskirchen , J. Lucifora , T. Michler , U. Protzer , Trends Pharmacol. Sci. 2014, 35, 470. 25108320 10.1016/j.tips.2014.06.004 PMC7112871

[8] D. P. Melendez , R. R. Razonable , Infect. Drug Resist. 2015, 8, 269. 26345608 10.2147/IDR.S79131 PMC4531042

[9] G. Maarifi , M. F. Martin , A. Zebboudj , A. Boulay , P. Nouaux , J. Fernandez , J. Lagisquet , D. Garcin , R. Gaudin , N. J. Arhel , S. Nisole , Cell Chem. Biol. 2022, 29, 1113. 35728599 10.1016/j.chembiol.2022.05.009 PMC9213012

[10] Z. A. Ratan , F. R. Mashrur , A. P. Chhoan , S. M.d. Shahriar , M. F. Haidere , N. J. Runa , S. Kim , D.‐H. Kweon , H. Hosseinzadeh , J. Y. Cho , Pharmaceutics 2021, 13, 2034. 34959320 10.3390/pharmaceutics13122034 PMC8705988

[11] M. Rai , S. D. Deshmukh , A. P. Ingle , A. K. Gade , Crit. Rev. Microbiol. 2016, 112, 46.

[12] K. A. Altammar , Front. Microbiol. 2023, 14, 1155622. 37180257 10.3389/fmicb.2023.1155622 PMC10168541

[13] Y. Wang , Y. Han , D. X. Xu , Environ. Sci. Ecotechnol. 2024, 19, 100325. 38046179 10.1016/j.ese.2023.100325 PMC10692670

[14] S. H. Lee , B. H. Jun , Int. J. Mol. Sci. 2019, 20, 865.30781560

[15] A. Gibała , P. Żeliszewska , T. Gosiewski , A. Krawczyk , D. Duraczyńska , J. Szaleniec , M. Szaleniec , M. Oćwieja , Biomolecules 2021, 11, 1481. 34680114 10.3390/biom11101481 PMC8533414

[16] Y. Qing , L. Cheng , R. Li , G. Liu , Y. Zhang , X. Tang , J. Wang , H.e Liu , Y. Qin , Int. J. Nanomed. 2018, 2018, 3311. 10.2147/IJN.S165125 PMC5993028 29892194

[17] J. Butler , R. D. Handy , M. Upton , A. Besinis , ACS Nano 2023, 17, 7064. 37027838 10.1021/acsnano.2c12488 PMC10134505

[18] D. de Lacerda Coriolano , J. Barbosa De Souza , E. V. Bueno , S. M. de Fátima Ramos dos Santos Medeiros , I. Dillion Lima Cavalcanti , I. Macário Ferro Cavalcanti , Braz. J. Microbiol. 2020, 267. 33231865 10.1007/s42770-020-00406-x PMC7966632

[19] C. Liao , Y. Li , S. C. Tjong , Int. J. Mol. Sci. 2019, 20, 449. 30669621 10.3390/ijms20020449 PMC6359645

[20] E. O. Mikhailova , J. Funct. Biomater. 2020, 11, 84. 33255874 10.3390/jfb11040084 PMC7711612

[21] J. L. Elechiguerra , J. L. Burt , J. R. Morones , A. Camacho‐Bragado , X. Gao , H. H. Lara , M. J. Yacaman , J. Nanobiotechnol. 2005, 3, 6. 10.1186/1477-3155-3-6 PMC1190212 15987516

[22] U. Ghosh , K. Sayef Ahammed , S. Mishra , A. Bhaumik , Chem. Asian J. 2022, 17, 202101149. 10.1002/asia.202101149 PMC9011828 35020270

[23] S. Galdiero , A. Falanga , M. Vitiello , M. Cantisani , V. Marra , M. Galdiero , Molecules 2011, 16, 8894. 22024958 10.3390/molecules16108894 PMC6264685

[24] A. Luceri , R. Francese , D. Lembo , M. Ferraris , C. Balagna , Microorganisms 2023, 11, 629. 36985203 10.3390/microorganisms11030629 PMC10056906

[25] M. Javanian , M. Barary , S. Ghebrehewet , V. Koppolu , V. K. R. Vasigala , S. Ebrahimpour , J. Med. Virol. 2021, 93, 4638. 33792930 10.1002/jmv.26990

[26] T. Flerlage , D. F. Boyd , V. Meliopoulos , P. G. Thomas , S. Schultz‐Cherry , Nat. Rev. Microbiol. 2021, 19, 425. 33824495 10.1038/s41579-021-00542-7 PMC8023351

[27] N. M. Bouvier , P. Palese , Vaccine 2008, 26, D49. 19230160 10.1016/j.vaccine.2008.07.039 PMC3074182

[28] M. Zhao , L. Wang , S. Li , Int. J. Mol. Sci. 2017, 18, 1673.28763020

[29] D. Dou , R. Revol , H. Östbye , H. Wang , R. Daniels , Front. Immunol. 2018, 9, 1581. 30079062 10.3389/fimmu.2018.01581 PMC6062596

[30] J. L. McAuley , B. P. Gilbertson , S. Trifkovic , L. E. Brown , J. L. McKimm‐Breschkin , Front. Microbiol. 2019, 10, 39. 30761095 10.3389/fmicb.2019.00039 PMC6362415

[31] R. Manzoor , M. Igarashi , A. Takada , Int. J. Mol. Sci. 2017, 18, 2649. 29215568 10.3390/ijms18122649 PMC5751251

[32] M. K. Safo , F. N. Musayev , P. D. Mosier , Q. Zhou , H. Xie , U. R. Desai , PLoS One 2014, 9, 109. 10.1371/journal.pone.0109510 PMC4190115 25295515

[33] Y. Hu , H. Sneyd , R. Dekant , Curr. Top. Med. Chem. 2017, 2271. 28240183 10.2174/1568026617666170224122508 PMC5967877

[34] C. Zhao , J. Pu , Viruses 2022, 14, 2141.36298694

[35] Z. Zhu , H. Fan , E. Fodor , PLoS Biol. 2023, 3002. 10.1371/journal.pbio.3002370 PMC10662765 37943954

[36] J. Blümel , R. Burger , C. Drosten , A. Gröner , L. Gürtler , M. Heiden , M. Hildebrandt , B. Jansen , H. Klamm , T. Montag‐Lessing , R. Offergeld , G. Pauli , R. Seitz , U. Schlenkrich , V. Schottstedt , H. Willkommen , C.‐H. Wirsing von König , Transf. Med. Hemother. 2009, 35, 32. 10.1159/000111480 PMC3083280 21547110

[37] A. C. Perofsky , J. Huddleston , C. Hansen , J. R. Barnes , T. Rowe , X. Xu , R. Kondor , D. E. Wentworth , N. Lewis , L. Whittaker , B. Ermetal , R. Harvey , M. Galiano , R. Stuart Daniels , J. W. McCauley , S. Fujisaki , K. Nakamura , N. Kishida , S. Watanabe , H. Hasegawa , S. G. Sullivan , I. G. Barr , K. Subbarao , F. Krammer , T. Bedford , C. Viboud , medRxiv 2023, 23, 296.

[38] J. Jeevanandam , S. Krishnan , Y. S. Hii , S. Pan , Y. S. Chan , C. Acquah , M. K. Danquah , J. Rodrigues , J. Nanostruct. Chem. 2022, 12, 809. 10.1007/s40097-021-00465-y PMC8760111 35070207

[39] P. Mehrbod , A. Ideris , A. R. Omar , M. Hair‐Bejo , Afr. J. Microbiol. Res. 2012, 6, 5715.

[40] D.‐X. Xiang , Q. Chen , L. Pang , C.‐L. Zheng , J. Virol. Methods. 2011, 178, 137. 21945220 10.1016/j.jviromet.2011.09.003

[41] Y. Mori , T. Ono , Y. Miyahira , V. Q. Nguyen , T. Matsui , M. Ishihara , Nanoscale Res. Lett. 2013, 8, 93. 23421446 10.1186/1556-276X-8-93 PMC3606407

[42] T. Joseph , D. Kar Mahapatra , A. Esmaeili , L. Piszczyk , M. Hasanin , M. Kattali , J. Haponiuk , S. Thomas , Nanomaterials 2023, 13, 574. 36770535 10.3390/nano13030574 PMC9920911

[43] Y. Li , Z. Lin , M. Zhao , M. Guo , T. T. Xu , C. B. Wang , H. M. Xia , B. Zhu , RSC Adv. 2016, 6, 89679.

[44] Y. Li , Z. Lin , M. Zhao , T. Xu , C. Wang , L. Hua , H. Wang , H. Xia , B. Zhu , ACS Appl. Mater. Interfaces 2016, 8, 24385. 27588566 10.1021/acsami.6b06613

[45] Z. Lin , Y. Li , M. Guo , T. Xu , C. Wang , M. Zhao , H. Wang , T. Chen , B. Zhu , RSC Adv. 2017, 7, 742.

[46] I. V. Kiseleva , F. M. Al , E. A. Skomorokhova , A. R. Rekstin , E. A. Bazhenova , D. N. Magazenkova , I. A. Orlov , L. G. Rudenko , M. Broggini , L. V. Puchkova , Vaccines 2020, 8, 679. 33202939 10.3390/vaccines8040679 PMC7712555

[47] Z. K. Alghrair , D. G. Fernig , B. Ebrahimi , Beilstein J. Nanotechnol. 2019, 10, 1038. 31165030 10.3762/bjnano.10.104 PMC6541353

[48] M. Fatima , N.‐U‐S. Sadaf Zaidi , D. Amraiz , F. Afzal , J. Microbiol. Biotechnol. 2016, 26, 151. 26403820 10.4014/jmb.1508.08024

[49] A. Ali , F. Hussain , S. Attacha , et al., Nanomaterials 2021, 2076. 34443906 10.3390/nano11082076 PMC8402186

[50] M. Saadh , Saudi J. Biol. Sci. 2021, 28, 6674. 34764780 10.1016/j.sjbs.2021.07.035 PMC8568804

[51] D. Srisrimal , S. Krishnan , A. Thirunavukkarasu , Lett. Appl. NanoBioSci. 2023.

[52] S. Seino , Y. Imoto , T. Kosaka , et al., MRS Adv. 2016, 705.

[53] S. Seino , Y. Ohkubo , T. Magara , H. Enomoto , E. Nakajima , T. Nishida , Y. Imoto , T. Nakagawa , Nanomaterials 2022, 12, 3046. 36080083 10.3390/nano12173046 PMC9457845

[54] K. Naumenko , S. Zahorodnia , C. V. Pop , N. Rizun , J. Virus Eradic. 2023, 9, 100330. 10.1016/j.jve.2023.100330 PMC10319835 37416089

[55] D. Xiang , Y. Zheng , W. Duan , et al., Int. J. Nanomed. 2013, 4103. 10.2147/IJN.S53622 PMC3817021 24204140

[56] N. Reuter , X. Chen , B. Kropff , A. S. Peter , W. J. Britt , M. Mach , K. Überla , M. Thomas , Viruses 2023, 15, 1500. 37515187 10.3390/v15071500 PMC10384293

[57] C. B. Jackson , M. Farzan , B. Chen , et al., Nat. Rev. Mol. Cell Biol. 2022, 23, 3. 34611326 10.1038/s41580-021-00418-x PMC8491763

[58] S. Lenhard , S. Gerlich , A. Khan , S. Rödl , J.‐E. Bökenkamp , E. Peker , C. Zarges , J. Faust , Z. Storchova , M. Räschle , J. Riemer , J. M. Herrmann , J. Cell Biol. 2023, 222, e202303002. 37682539 10.1083/jcb.202303002 PMC10491932

[59] A. Kwiatkowska , L. H. Granicka , Membranes 2023, 13, 464. 37233525 10.3390/membranes13050464 PMC10223398

[60] A. Salleh , R. Naomi , N. D. Utami , et al., Nanomaterials 2020, 10, 1566. 32784939 10.3390/nano10081566 PMC7466543

[61] A. Kumar , K. Nath , Y. Parekh , et al., Colloid Interface Sci. Commun. 2021, 100542. 34729365 10.1016/j.colcom.2021.100542 PMC8554045

[62] C. Balagna , S. Perero , E. Percivalle , et al., Open Ceramics. 2020, 100 006.

[63] C. Estevan , E. Vilanova , M. A. Sogorb , Arch. Toxicol. 2022, 96, 105. 34786588 10.1007/s00204-021-03187-w PMC8594636

[64] M. Baselga , I. Uranga‐Murillo , D. de Miguel , et al., Materials 2022, 15, 4742. 35888208 10.3390/ma15144742 PMC9318907

[65] S. S. Jeremiah , K. Miyakawa , T. Morita , et al., Biochem. Biophys. Res. Commun. 2020, 533, 195. 32958250 10.1016/j.bbrc.2020.09.018 PMC7486059

[66] Q. He , J. Lu , N. Liu , W. Lu , Y.u Li , C. Shang , X. Li , L. Hu , G. Jiang , Nanomaterials 2022, 12, 990. 35335803 10.3390/nano12060990 PMC8950764

[67] P. Merkl , S. Long , G. M. McInerney , et al., Nanomaterials 2021, 17, 1312. 10.3390/nano11051312 PMC8155969 34067553

[68] D. Asmat‐Campos , J. Rojas‐Jaimes , G. M. de Oca‐Vásquez , et al., Nat. Res. 2023, 13, 9772. 10.1038/s41598-023-36910-x PMC10275893 37328549

[69] W. T. Lam , T. S. Babra , J. H. D. Smith , et al., Polymers 2022, 14, 4172.36236120

[70] R. Díaz‐Puertas , E. Rodríguez‐Cañas , M. Bello‐Perez , M. Fernández‐Oliver , R. Mallavia , A. Falco , Nanomaterials 2023, 13, 1467. 37177014 10.3390/nano13091467 PMC10180066

[71] D. J. da Silva , G. B. Gramcianinov , P. Z. Jorge , et al., Front. Chem. 2023, 11, 1083. 10.3389/fchem.2023.1083399 PMC10042293 36993814

[72] H. Almanza‐Reyes , S. Moreno , I. Plascencia‐López , et al., PLoS One 2021, 16, e0256401. 34411199 10.1371/journal.pone.0256401 PMC8375774

[73] L. Wieler , O. Vittos , N. Mukherjee , et al., Heliyon 2023, 9, 14419. 10.1016/j.heliyon.2023.e14419 PMC10008037 36942214

[74] G. Gupta , B. Hamawandi , D. J. Sheward , et al., Front. Bioeng. Biotechnol. 2022, 10, 1083232. 36578508 10.3389/fbioe.2022.1083232 PMC9790969

[75] D. Stanisic , G. C. F. Cruz , L. A. Elias , et al., Front. Bioeng. Biotechnol. 2022, 10, 858156. 35646854 10.3389/fbioe.2022.858156 PMC9133937

[76] A. Z. Medvedev , B. B. Bokhonov , O. S. Kiselev , et al., Mater. Lett. 2023, 346, 134557. 37215536 10.1016/j.matlet.2023.134557 PMC10192065

[77] O. V. Morozova , V. A. Manuvera , A. E. Grishchechkin , N. A. Barinov , N. V. Shevlyagina , V. G. Zhukhovitsky , V. N. Lazarev , D. V. Klinov , Viruses 2022, 14, 902. 35632644 10.3390/v14050902 PMC9144282

[78] M. M. Al‐Sanea , N. Abelyan , M. A. Abdelgawad , A. Musa , M. M. Ghoneim , T. Al‐Warhi , N. Aljaeed , O. J. Alotaibi , T. S. Alnusaire , S. F. Abdelwahab , A. Helmy , U. R. Abdelmohsen , K. A. Youssif , Antibiotics 2021, 10, 824. 34356745 10.3390/antibiotics10070824 PMC8300822

[79] D. Srisrimal , S. Krishnan , A. Thirunavukkarasu , Lett. Appl. NanoBioSci. 2023, 4, 1.

[80] I. Ahmad , D. W. Wilson , Int. J. Mol. Sci. 2020, 21, 5969. 32825127 10.3390/ijms21175969 PMC7503644

[81] A. Mohammed Fayaz , Z. Ao , M. Girilal , et al., Int. J. Nanomed. 2012, 5, 5007. 10.2147/IJN.S34973 PMC3459690 23049252

[82] R. L. Hu , S. R. Li , F. J. Kong , R. J. Hou , X. L. Guan , F. Guo , Genet. Mol. Res. 2014, 7022. 24682984 10.4238/2014.March.19.2

[83] M. V. Morone , A. Chianese , F. Dell'Annunziata , V. Folliero , E. P. Lamparelli , G. Della Porta , C. Zannella , A. De Filippis , G. Franci , M. Galdiero , A. Morone , Microorganisms 2024, 12, 820. 38674764 10.3390/microorganisms12040820 PMC11052337

[84] P. Orlowski , E. Tomaszewska , M. Gniadek , P. Baska , J. Nowakowska , J. Sokolowska , Z. Nowak , M. Donten , G. Celichowski , J. Grobelny , M. Krzyzowska , PLoS One 2014, e104113. 25117537 10.1371/journal.pone.0104113 PMC4130517

[85] E. Szymanska , P. Orlowski , K. Winnicka , E. Tomaszewska , P. Baska , G. Celichowski , J. Grobelny , A. Basa , M. Krzyzowska , Int. J. Mol. Sci. 2018, 19, 387. 29382085 10.3390/ijms19020387 PMC5855609

[86] R. S. El‐Mohamady , T. A. Ghattas , M. F. Zawrah , Y. G. M. Abd El‐Ha , Int. J. Vet. Sci. Med. 2018, 2, 296. 10.1016/j.ijvsm.2018.09.002 PMC6286414 30564612

[87] M. Krzyzowska , M. Chodkowski , M. Janicka , D. Dmowska , E. Tomaszewska , K. Ranoszek‐Soliwoda , K. Bednarczyk , G. Celichowski , J. Grobelny , Microorganisms 2022, 10, 110. 35056558 10.3390/microorganisms10010110 PMC8780146

[88] M. Krzyzowska , M. Janicka , M. Chodkowski , M. Patrycy , O. Obuch‐Woszczatyńska , E. Tomaszewska , K. Ranoszek‐Soliwoda , G. Celichowski , J. Grobelny , Viruses 2023, 15, 2024. 37896801 10.3390/v15102024 PMC10611064

[89] S. Gaikwad , A. Ingle , A. Gade , et al., Int. J. Nanomed. 2013, 4303. 10.2147/IJN.S50070 PMC3826769 24235828

[90] G. S. G. Zeedan , K. A. Abd El‐Razik , A. M. Allam , et al., Adv. Anim. Vet. Sci. 2020, 433.

[91] A. Dhanasezhian , S. Srivani , K. Govindaraju , et al., Ind. J. GeoMarine Sci. 2019, 1252.

[92] E. Haggag , A. Elshamy , M. Rabeh , N. Gabr , M. Salem , K. Youssif , A. Samir , A. Bin Muhsinah , A. Alsayari , U. R. Abdelmohsen , Int. J. Nanomed. 2019, 14, 6217. 10.2147/IJN.S214171 PMC6690046 31496682

[93] M. A. Ramadan , A. E. Shawkey , M. A. Rabeh , et al., J. Herbal Med. 2020, 100 289.

[94] M. Hasanin , M. A. Elbahnasawy , A. M. Shehabeldine , A. H. Hashem , BioMetals 2021, 34, 1313. 34564808 10.1007/s10534-021-00344-7 PMC8475443

[95] M. M. El‐Sheekh , M. T. Shabaan , L. Hassan , et al., Int. J. Environ. Health Res. 2020, 32, 616. 32627584 10.1080/09603123.2020.1789946

[96] M. M. El‐Sheekh , A. Mohamed , S. S. El , et al., Egypt. J. Aquatic Biol. Fisheries 2022, 213.

[97] R. Seitz , Transf. Med. Hemother. 2016, 203.

[98] Y. van Heuvel , S. Schatz , J. F. Rosengarten , J. Stitz , Toxins 2022, 14, 138. 35202165 10.3390/toxins14020138 PMC8876946

[99] C. B. Wilen , J. C. Tilton , R. W. Doms , Cold Spring. Harb. Perspect. Med. 2012, 2, a006866. 22908191 10.1101/cshperspect.a006866 PMC3405824

[100] H. H. Lara , N. V. Ayala‐Nuñez , L. Ixtepan‐Turrent , C. Rodriguez‐Padilla , J. Nanobiotechnol. 2010, 8, 1. 10.1186/1477-3155-8-1 PMC2818642 20145735

[101] H. H. Lara , L. Ixtepan‐Turrent , E. N. Garza‐Treviño , C. Rodriguez‐Padilla , J. Nanobiotechnol. 2010, 8, 15. 10.1186/1477-3155-8-15 PMC2911397 20626911

[102] H. H. Lara , L. Ixtepan‐Turrent , E. N. Garza Treviño , et al., J. Nanobiotechnol. 2011, 9, 38. 10.1186/1477-3155-9-38 PMC3180349 21923937

[103] M. S. Ardestani , A. S. Fordoei , A. Abdoli , et al., J. Mater. Sci.: Mater. Med. 2015, 26, 179. 25893388 10.1007/s10856-015-5510-7

[104] S. D. Kumar , G. Singaravelu , S. Ajithkumar , K. Murugan , M. Nicoletti , G. Benelli , J. Cluster Sci. 2017, 28, 359.

[105] R. K. Sharma , K. Cwiklinski , R. Aalinkeel , et al., Immunol. Invest. 2017, 46, 833. 29058549 10.1080/08820139.2017.1371908

[106] Y. Sampathkumar , E. Sanniyasi , M. Priya Arumugam , et al., World J. Adv. Pharma. Life Sci. 2022, 024.

[107] A. Murugesan , M. Manoharan , Emerging and Reemerging Viral Pathogens, Academic Press, Cambridge, MA 2019, 281.

[108] M. G. Guzman , D. J. Gubler , A. Izquierdo , et al., Nat. Rev. Dis. Primers 2016, 16055. 27534439 10.1038/nrdp.2016.55

[109] G. Benelli , Enzyme Microb. Technol. 2016, 95, 58. 27866627 10.1016/j.enzmictec.2016.08.022

[110] V. Sujitha , K. Murugan , M. Paulpandi , C. Panneerselvam , U. Suresh , M. Roni , M. Nicoletti , A. Higuchi , P. Madhiyazhagan , J. Subramaniam , D. Dinesh , C. Vadivalagan , B. Chandramohan , A. A. Alarfaj , M. A. Munusamy , D. R. Barnard , G. Benelli , Parasitol. Res. 2015, 114, 3315. 26063530 10.1007/s00436-015-4556-2

[111] K. Murugan , D. Dinesh , M. Paulpandi , A. D. M. Althbyani , J. Subramaniam , P. Madhiyazhagan , L. Wang , U. Suresh , P. M. Kumar , J. Mohan , R. Rajaganesh , H. Wei , K. Kalimuthu , M. N. Parajulee , H. Mehlhorn , G. Benelli , Parasitol. Res. 2015, 114, 4349. 26290219 10.1007/s00436-015-4676-8

[112] K. Murugan , C. Panneerselvam , J. Subramaniam , et al., Environ. Sci. Pollution Res. 2016, 23, 16671. 10.1007/s11356-016-6832-9 27180838

[113] P. Patil , K. Alagarasu , D. Chowdhury , et al., Heliyon. 2022, 30, 11879. 10.1016/j.heliyon.2022.e11879 PMC9723942 36483307

[114] T. Thi Ngoc Dung , N. Thi Thu Thuy , V. T. B. Hau , et al., Nanotechnology 2023, 34. 10.1088/1361-6528/ac97a2 36198234

[115] R. Khan , H. Naureen , A. Javed , M. Khalid , H. Khan , Appl. Microbiol. Biotechnol. 2023, 107, 111. 36441209 10.1007/s00253-022-12298-y

[116] M. K. S. van Duijl‐Richter , T. E. Hoornweg , I. A. Rodenhuis‐Zybert , et al., Viruses. 2015, 3647. 26198242 10.3390/v7072792 PMC4517121

[117] V. Sharma , S. Kaushik , P. Pandit , D. Dhull , J. P. Yadav , S. Kaushik , Appl. Microbiol. Biotechnol. 2019, 103, 881. 30413849 10.1007/s00253-018-9488-1

[118] S. Choudhary , R. Kumar , U. Dalal , et al., Mater. Sci. Eng. C. 2020, 112, 110934. 10.1016/j.msec.2020.110934 32409081

[119] Y. Sharma , A. Kawatra , V. Sharma , et al., Virus Dis. 2021, 32, 250.

[120] L. Lu , R. Wai‐Yin Sun , R. Chen , et al., Antiviral Ther. 2008, 13, 253. 18505176

[121] N. H. Shady , A. R. Khattab , S. Ahmed , M. Liu , R. J. Quinn , M. A. Fouad , M. S. Kamel , A. B. Muhsinah , M. Krischke , M. J. Mueller , U. R. Abdelmohsen , Int. J. Nanomed. 2020, 15, 3377. 10.2147/IJN.S233766 PMC7231760 32494136

[122] J. V. Rogers , C. V. Parkinson , Y. W. Choi , J. L. Speshock , S. M. Hussain , Nanoscale Res. Lett. 2008, 3, 129.

[123] J. L. Speshock , R. C. Murdock , L. K. Braydich‐Stolle , A. M. Schrand , S. M. Hussain , J. Nanobiotechnol. 2010, 8, 19. 10.1186/1477-3155-8-19 PMC2936366 20718972

[124] J. C. Trefry , D. P. Wooley , J. Biomed. Nanotechnol. 2013, 9, 1624. 23980510 10.1166/jbn.2013.1659

[125] N. Khandelwal , G. Kaur , K. K. Chaubey , et al., Virus Res. 2014, 190, 1. 24979044 10.1016/j.virusres.2014.06.011

[126] X. X. Yang , C. M. Li , C. Z. Huang , Nanoscale 2016, 8, 3040. 26781043 10.1039/c5nr07918g

[127] D. Morris , M. Ansar , J. Speshock , T. Ivanciuc , Y. Qu , A. Casola , R. Garofalo , Viruses 2019, 11, 732. 31398832 10.3390/v11080732 PMC6723559

[128] B. Borrego , G. Lorenzo , J. D. Mota‐Morales , et al., Nanomedicine. 2016, 12, 1185. 26970026 10.1016/j.nano.2016.01.021

[129] Y.i‐N. Chen , Y.i‐H. Hsueh , C.‐T.e Hsieh , D.‐Y. Tzou , P.‐L. Chang , Int. J. Environ. Res. Public Health 2016, 13, 430. 27104546 10.3390/ijerph13040430 PMC4847092

[130] C. Wan , J. Tai , J. Zhang , et al., Cell Death Dis. 2019, 10, 392. 31113937 10.1038/s41419-019-1624-z PMC6529488

[131] M. Saadh , Arabian J. Chem. 2022, 15, 103899.

[132] T. T. N. Dung , V. N. Nam , T. T. Nhan , et al., Mater. Res. Express. 2019, 6, 1250g9.

[133] M. García‐Serradilla , C. Risco , Virus Res. 2021, 302, 198444. 33961898 10.1016/j.virusres.2021.198444

[134] T. Hamouda , H. M. Ibrahim , H. H. Kafafy , H. M. Mashaly , N. H. Mohamed , N. M. Aly , Int. J. Biol. Macromol. 2021, 181, 990. 33864870 10.1016/j.ijbiomac.2021.04.071 PMC8056979

[135] E. H. Elshazly , A. Nasr , M. E. Elnosary , G. A. Gouda , H. Mohamed , Y. Song , Molecules 2023, 28, 1375. 36771041 10.3390/molecules28031375 PMC9919260

[136] M. J. Saadh , Arch. Virol. 2023, 32. 36604362 10.1007/s00705-022-05667-5

[137] M. J. Saadh , Int. J. Appl. Pharm. 2023, 163.

[138] S. Iravani , H. Korbekandi , S. V. Mirmohammadi , et al., Res. Pharm. Sci. 2014, 6, 385. PMC4326978 26339255

[139] J. Palanisamy , V. S. Palanichamy , G. Vellaichamy , P. Perumal , J. Vinayagam , S. Gunalan , et al., Naunyn Schmiedeberg's Arch. Pharmacol. 2024. 10.1007/s00210-024-03547-0 39560753

[140] P. Bélteky , A. Rónavári , N. Igaz , B. Szerencsés , I. Y. Tóth , I. Pfeiffer , M. Kiricsi , Z. Kónya , Int. J. Nanomed. 2019, 14, 667. 10.2147/IJN.S185965 PMC6342213 30705586

[141] P. M. Potter , J. Navratilova , K. R. Rogers , et al., Environ. Sci. Nano 2019, 2, 592. PMC6854907 31728194

[142] S. Xiong , X. Cao , H. Fang , et al., Sci. Total Environ. 2021, 775. 10.1016/j.scitotenv.2021.145867 33621870

[143] T. Bruna , F. Maldonado‐Bravo , P. Jara , N. Caro , Int. J. Mol. Sci. 2021, 22, 7202. 34281254 10.3390/ijms22137202 PMC8268496

[144] J. H. Shannahan , R. Podila , A. A. Aldossari , H. Emerson , B. A. Powell , P.u C. Ke , A. M. Rao , J. M. Brown , Toxicol. Sci. 2015, 143, 136. 25326241 10.1093/toxsci/kfu217 PMC4274384

[145] L. Xu , Y. Y. Wang , J. Huang , et al., Theranostics. 2020, 10, 8996. 32802176 10.7150/thno.45413 PMC7415816

[146] P. R. More , S. Pandit , A. D.e Filippis , G. Franci , I. Mijakovic , M. Galdiero , Microorganisms 2023, 11, 369. 36838334 10.3390/microorganisms11020369 PMC9961011

[147] C. Kunst , S. Schmid , M. Michalski , D. Tümen , J. Buttenschön , M. Müller , K. Gülow , Biomedicines 2023, 11, 1388. 37239059 10.3390/biomedicines11051388 PMC10216031

[148] S. Peixoto , S. Loureiro , I. Henriques , J. Hazard. Mater. 2022, 422, 126793. 34399213 10.1016/j.jhazmat.2021.126793

[149] X.‐L. Wang , N. Yu , C. Wang , H.‐R. Zhou , C. Wu , L. Yang , S.i Wei , A.i‐J. Miao , ACS Nano 2022, 16, 19002. 36315867 10.1021/acsnano.2c07924

[150] J. Ni , Z. Yang , Y. Zhang , et al., Front. Microbiol. 2022, 13, 1048216. 36569079 10.3389/fmicb.2022.1048216 PMC9772453

[151] X. Wang , X. Cui , J. Wu , et al., J. Mater. Chem. B. 2023, 11, 1904. 36734837 10.1039/d2tb02756a

[152] B. Lamas , L. Evariste , E. Houdeau , Environ. Pollution 2023, 330, 121795. 10.1016/j.envpol.2023.121795 37187281

[153] Z. Ferdous , A. Nemmar , Int. J. Mol. Sci. 2020, 21, 2375. 32235542 10.3390/ijms21072375 PMC7177798

[154] L. Strużyńska , Int. J. Mol. Sci. 2023, 24, 15386. 37895066 10.3390/ijms242015386 PMC10607027

[155] S. Gurunathan , M. Jeyaraj , M.‐H. Kang , J.‐H. Kim , Int. J. Mol. Sci. 2019, 20, 4439. 30634552 10.3390/ijms20020247 PMC6359521

[156] Z. Yu , Q. Li , J. Wang , Y. Yu , Y. Wang , Q. Zhou , P. Li , Nanoscale Res. Lett. 2020, 15, 115. 32436107 10.1186/s11671-020-03344-7 PMC7239959

[157] B. Dabrowska‐Bouta , G. Sulkowski , M. Gewartowska , L. Struzynska , Int. J. Mol. Sci. 2022, 23, 13013. 36361797 10.3390/ijms232113013 PMC9655133

[158] I. Florance , M. Cordani , P. Pashootan , et al., Cell. Mol. Life Sci. 2024, 17, 184. 10.1007/s00018-024-05199-y PMC11024050 38630152

[159] S. Sampath , A. Khedr , S. Qamar , et al., Cureus. 2021, 13, e18136. 34692344 10.7759/cureus.18136 PMC8525686

[160] A. A. Adalja , M. Watson , E. S. Toner , et al., Curr. Top. Microbiol. Immunol. 2019, 424, 1. 31463536 10.1007/82_2019_176 PMC7122301

[161] P. Orlowski , A. Kowalczyk , E. Tomaszewska , K. Ranoszek‐Soliwoda , A. Wegrzyn , J. Grzesiak , G. Celichowski , J. Grobelny , K. Eriksson , M. Krzyzowska , Viruses 2018, 10, 524. 30261662 10.3390/v10100524 PMC6213294

[162] Y. Li , Z. Lin , M. Guo , M. Zhao , Y.u Xia , C. Wang , T. Xu , B. Zhu , Int. J. Nanomed. 2018, 13, 2005. 10.2147/IJN.S155994 PMC5892959 29662313

[163] M. Noga , J. Milan , A. Frydrych , K. Jurowski , Int. J. Mol. Sci. 2023, 24, 5133. 36982206 10.3390/ijms24065133 PMC10049346

[164] S. Bohlooli , A. Mohebipoor , S. Mohammadi , M. Kouhnavard , S. Pashapoor , Int. J. Dermatol. 2007, 46, 524. 17472688 10.1111/j.1365-4632.2007.03159.x

[165] M. Dias , R. Zhang , T. Lammers , et al., Drug Deliv. Transl. Res. 2024, 15, 789. 39377875 10.1007/s13346-024-01716-5 PMC11782377

[166] C. R. Romo‐Quiñonez , A. R. Álvarez‐Sánchez , P. Álvarez‐Ruiz , et al., PeerJ 2020, 2020. 10.7717/peerj.8446 PMC7049459 32149020

[167] L. Xuan , Z. Ju , M. Skonieczna , et al., MedComm. 2023. 10.1002/mco2.327 PMC10349198 37457660

